# A Heterogenized Copper Phenanthroline System to Catalyze the Oxygen Reduction Reaction

**DOI:** 10.1002/celc.202101365

**Published:** 2022-02-02

**Authors:** Cornelis J. M. van der Ham, Damy N. H. Zwagerman, Longfei Wu, Jan P. Hofmann, Dennis G. H. Hetterscheid

**Affiliations:** ^1^ Leiden Institute of Chemistry P.O. Box 9502 2300 RA Leiden the Netherlands; ^2^ Laboratory for Inorganic Materials and Catalysis De partment of Chemical Engineering and Chemistry Eindhoven University of Technology P.O. Box 513 5600 MB Eindhoven The Netherlands; ^3^ Current address: Inorganic Chemistry and Catalysis, Department of Chemistry Utrecht University Universiteitsweg 99 3584 CG Utrecht The Netherlands; ^4^ Current address: Surface Science Laboratory, Department of Materials and Earth Sciences Technical University of Darmstadt Otto-Berndt-Strasse 3 64287 Darmstadt Germany

**Keywords:** Copper, Diazocoupling, Electrochemistry, Oxygen Reduction, Surface Immobilization

## Abstract

Upon the electrochemical reduction of an in situ generated 5‐diazo‐1,10‐phenanthroline ion, phenanthroline was covalently attached to a gold electrode. The grafted molecules act as a ligand when brought in contact with a copper‐containing electrolyte solution. As the ligands are limited in spatial movement, the exclusive formation of the active species with only one phenanthroline ligand coordinated was expected. The in situ generated complexes have been investigated for activity in the oxygen reduction reaction, for which an overpotential of 800 mV is observed. During catalysis, initially a thick copper layer is formed on top of an organic layer that is still present on the gold surface. Upon deterioration of the organic layer underneath the copper over time, the amount of copper on the electrode and thereby the electrocatalytic activity decreases.

## Graphical abstract

**Beneficial layer**: The immobilization of phenanthroline via a diazonium coupling reaction leads to a well‐defined thin organic layer on gold. Electrochemical reduction in the presence of Cu^2+^ results in a relatively thick layer of copper, which is active for the oxygen reduction reaction. The activity depends on the presence of both copper and the organic ligand.

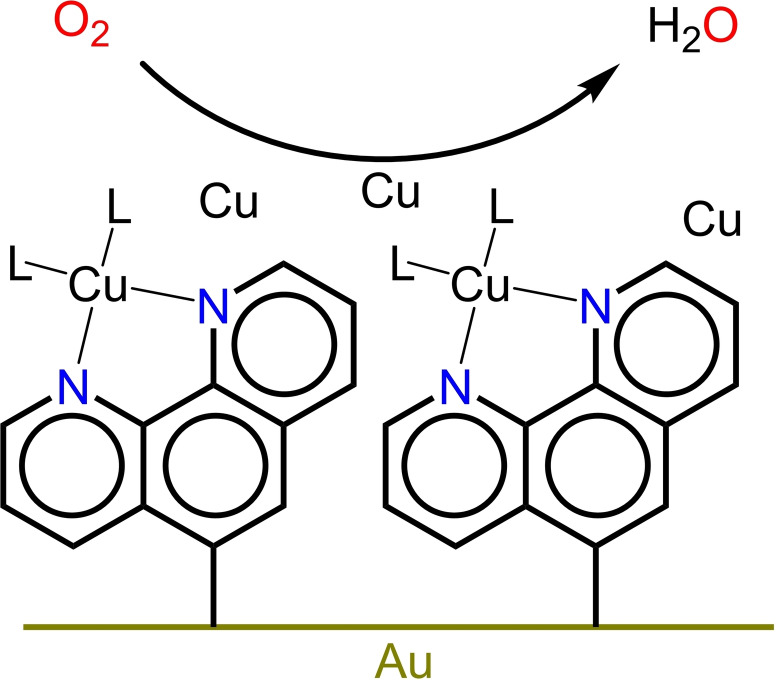

## 1 Introduction

The oxygen reduction reaction is of interest for the development of efficient fuel cells wherein it is the counter reaction to the hydrogen oxidation reaction. In order to find an optimally working catalyst for the oxygen reduction reaction, the binding energies of all intermediates of the oxygen reduction cycle need to be optimized simultaneously. This will result in an ideal potential energy landscape wherein all redox intermediates within the catalytic cycle are found at the equilibrium potential of water. Under these conditions, catalysis should be feasible at the equilibrium potential of the water oxidation/oxygen reduction reactions. Since the various catalytic intermediates bind in a very similar manner to a heterogeneous surface, it is not possible to align these to the same potential energy. This inability to align the catalytic intermediates from an energetic point of view is referred to as a scaling relationship, first described by Nørskov et al.^[1]^ Due to such scaling relations the oxygen reduction reaction has a theoretical minimum overpotential of 0.41 V at a metal surface.^[2]^

In nature, laccase is an efficient copper‐based enzyme for the reduction of oxygen to water.^[3]^ Due to the complex protein scaffold the reaction pathway for the oxygen reduction reaction is different to that of heterogeneous catalysts. This difference in reaction mechanism leads to a different potential energy landscape and allows for a lower overpotential of the oxygen reduction reaction compared to heterogeneous surfaces. Inspired by multi‐copper enzymes such as laccase, activation of oxygen at molecular copper sites has been an important area of research in the bioinorganic chemistry community.^[4]^ The electrochemical reduction of oxygen mediated by molecular copper catalysts has received somewhat less attention, and typically only mediocre activities have been reported.^[5]^

Recently, we have reported the reduction of oxygen mediated by Cu(tmpa), which showed record breaking rates for oxygen reduction with a turnover frequency of 1.8×10^6^ s^−1^ (tmpa=tris(2‐pyridylmethyl)amine).^[6]^ The electrochemical reduction proceeds in a stepwise process wherein peroxide is detected as an isolable intermediate. During the rate determining step, oxygen binding occurs at a copper(I) species which results in formation of presumably a copper(II) superoxide. It is noteworthy that the reported catalytic rates match well with the binding constant of O_2_ to Cu^I^ in organic media.^[7]^ The overpotential for ORR is relatively high with 750 mV versus the reversible hydrogen electrode (RHE). Given that hydrogen peroxide is an obligatory intermediate that is formed via a 2+2 mechanism, the minimum overpotential for ORR is probably related to the equilibrium potential of hydrogen peroxide, found at 0.7 V versus RHE. To increase the potential where copper(I) formation becomes feasible and thereby to lower the overpotential for ORR and/or peroxide formation, copper systems with weaker N‐donor ligands are necessary.

Ligand exchange kinetics at copper sites are fast, and formation of free copper is likely to trigger deposition of copper(0) on the electrode interface,^[8]^ particularly when weakly coordinating ligands are employed. In search of control over the overpotential we opted for the bidentate phenanthroline (phen) framework, which has been explored previously by Anson et al.^[9]^ Ligand dissociation in this case was successfully countered by addition of excess ligand. Under these conditions exclusively Cu(phen)_2_ species were observed. In contrast, our preliminary studies wherein Cu is kept in solution by co‐solving it with phenanthroline in a chloride rich medium, showed that the mono‐ligated Cu(phen) is the active species that is capable of ORR catalysis. However, this species suffers from inhibition by addition of excess ligand and deposition of copper(0) on the electrode interface during electrochemical reduction.

**Figure 1:**
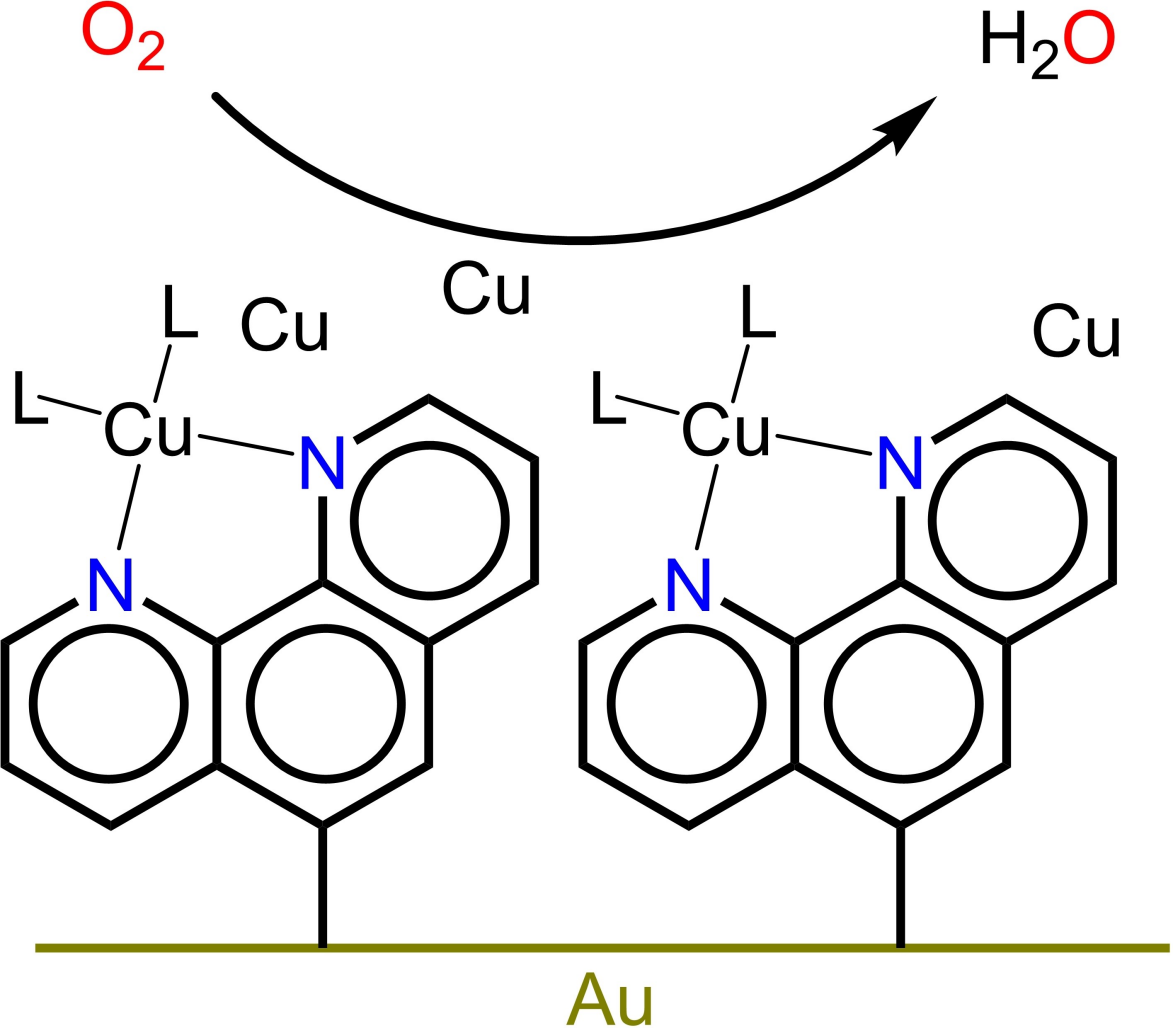
Schematic representations of the oxygen reduction reaction mediated with surface immobilized phenanthroline in the presence of a copper salt.

To interrogate this mono‐ligated Cu(phen) species in absence of copper(0) deposits we studied the ORR chemistry of surface immobilized phenanthroline copper species. By close packing of the phenanthroline ligands on gold using a diazonium coupling reaction of 1,10‐phenanthroline‐5‐diazonium, the direct deposition of copper(0) is hypothesized to be avoided, while considerable concentrations of copper(II) in solution can be maintained to prevent leaching of copper from the phenanthroline sites (Figure 1).

## 2 Results and Discussion

### 2.1 Voltammetry and EPR spectroscopy of in situ generated copper phenanthroline complexes in the homogeneous phase

Due to the fast ligand exchange kinetics of copper,^[10]^ bidentate ligands were expected to readily decoordinate, leading to small concentrations of free Cu^2+^. Non‐ligated copper species are likely to form copper(0) deposits on the electrode under oxygen reduction conditions, which should be avoided altogether.^[8a]^ To push the equilibrium involving the bidentate phenanthroline ligand and copper towards complex formation, high concentrations and high ligand to copper ratios were employed. The latter strategy does not necessarily ensure formation of a catalytically active species as saturation of the copper sites may occur in the presence of excess ligands. We therefore investigated the structure and electrochemical activity of copper phenanthroline complexes at varying ligand to copper ratios to find an optimum between activity and stability of the homogeneous sites. Cu^2+^ is insoluble in a perchlorate solution in the presence of a phosphate buffer and in absence of any ligand. To avoid precipitation of copper from the reaction mixture, an electrolyte based on chloride was selected, which ensured that all copper species remained in solution prior to the electrochemistry experiments.

Copper phenanthroline was formed in situ in solution by preparing an electrolyte solution with 1 mm Cu^II^ and 1 mm 1,10‐phenanthroline in 0.1 m phosphate buffer containing 0.05 m NaCl acidified to pH 4 using HCl. For this system no well‐defined redox couples were found (Figure S2). With an increasing ratio between 1,10‐phenanthroline and copper the Cu^I^/Cu^II^ redox couple became more reversible (Figure S3). At a ligand to copper ratio >10, the complex starts to behave like a homogeneous species in voltammetry experiments with good linearity between i_p_ and the square root of the scan rate according the Randles‐Ševcik equation [Equation (S1), Figure S6].

Simulation of EPR spectra that were recorded under equimolar copper to ligand ratios yield g‐values of $g_{⊥}$ =2.05 and g_∥_=2.25 and show that Cu(phen) most likely has a square pyramidal structure with a $d_{z^{2}}$ SOMO (see Figure S8, S9 and table S1).^[11]^ At a ligand to copper ratio of 2 : 1 exclusive formation of a new species, presumably Cu(phen)_2_, was observed by EPR. Simulation of the EPR spectrum yields $g_{⊥}$ =2.17 and g_∥_=2.03, which point to a trigonal bipyramidal structure with a $d_{x^{2}{-y}^{2}}$ SOMO (more details are given in the SI). The EPR spectrum of the latter species does not change any further upon addition of excess ligand. This suggests that a 2 : 1 ratio of ligand to copper is sufficient to produce almost exclusively Cu(phen)_2_, but that considerable larger amounts of phenanthroline are necessary to maintain the concentration of free Cu^2+^ sufficiently low to avoid copper deposits on the electrode surface. An apparent similar behavior was observed in case of the ligands DMP and bipyridine (Table S2).

Cyclic voltammetry results in the presence of O_2_ illustrate that only the Cu(phen) complex is active towards the ORR, whereas Cu(phen)_2_ is not. Most likely the preferred tetrahedral geometry of copper(I) prevents any efficient binding of O_2_ to Cu(phen)_2_. These results appear to be in line with results obtained by the Anson group who studied the oxygen reduction reaction with physisorbed copper complexes with phenanthroline based ligands on carbon electrodes.[^9^, ^12^] Under their conditions wherein multiple layers of complex are present on the carbon surface and where π‐stacking interactions presumably take an important role, catalytic activity was pinpointed to Cu(phen) species. The activity of Cu(phen) is substantially lower compared to Cu(tmpa), although accurate rate constants could not be obtained using either the‐foot‐of‐the‐wave or other current‐enhancement methods^[13]^ due to the irreversible behavior of the Cu^II^/Cu^I^ reduction wave. It is noteworthy that the background activity of non‐ligated Cu^2+^ and its deposits do not show significant ORR activity with sodium chloride as an electrolyte (Figure S7).

### 2.2 The reduction of diazonium ions for the covalent coupling of organic molecules to electrode surfaces

To break the apparent deadlock between copper deposition at low ligand to copper ratios and the lack of activity at high ligand to copper ratios we investigated the electrochemistry of surface immobilized phenanthroline on gold. Gold was chosen here over other electrode materials, as it allows for a full coverage of the electrode with organic functionalities through a diazonium coupling reaction.[^14^, ^15^] Within this strategy full coverage of the electrode surface with immobilized phenanthroline would only allow for catalysis via the phenanthroline framework, whereas small concentrations of Cu^2+^ in solution would ensure that (most of) the phenanthroline would bind copper.

An advantage of the immobilization of **1** via the reduction of a diazonium ion is that it is known to form thin layers on the electrode.^[14]^ Upon the reduction of 5‐diazo‐1,10‐phenanthroline (**1**‐N_2_^+^ ) at glassy carbon electrodes an organic layer with a thickness of 2 nm is formed.^[15]^ Immobilization of **1** was also performed by the reduction of 5‐bromo‐1,10‐phenanthroline.^[16]^ The thickness of the layer formed upon the reduction of 5‐bromo‐1,10‐phenanthroline is 125 nm, which is much thicker than the layer formed by the reduction of **1**‐N_2_^**+**^. Also, the reduction of 1,10‐phenanthroline itself leads to formation of an organic layer on the electrode. This direct reduction of 1,10‐phenanthroline at glassy carbon electrodes happens at adsorbed 1,10‐phenanthroline molecules via a proton coupled electron transfer step which leads to the formation of a carbon centered radical at the 4‐position. The radical couples with the glassy carbon electrode resulting in the loss of aromaticity on one of the phenyl rings. The thickness of the organic layer on the electrode is approximately 2 nm, similar to the layer formed by the reduction of **1**‐N_2_^**+**^.^[15]^ We anticipate that the geometry of the molecules in the case of reduction of **1**‐N_2_^**+**^ at the electrode surface is more advantageous for obtaining a square pyramidal mono ligated structure than starting from 1,10‐phenanthroline.

The proposed mechanism of the reduction of diazonium ions proceeds via the formation of radicals. Such radicals are highly reactive and consequently easily dimerize, forming diamagnetic dimers which themselves do not attach to the electrode surface. It is also possible that the radicals react with molecules which are already attached to the electrode surface, forming thicker layers of organic material on the electrode surface. This has been observed in the immobilization of **1** on glassy carbon electrodes.^[15]^ Analysis of Atomic Force Microscopy (AFM) studies of the grafted layer points to a layer thickness of 2 nm, which is more than twice the length of a phenanthroline molecule in the gas phase. The generated radicals may also react with residual oxygen. In summary, these side reactions mean that the efficiency of the immobilization reaction can be rather low. Nevertheless obtaining a full coverage of the gold surface with organic molecules via the diazonium coupling has been shown previously.[^14^, ^15^] We anticipate that these layers allow to have small concentrations of copper in the electrolyte without obtaining direct deposition of copper on gold under reductive conditions (see Figure 1). Within this approach, 4‐bromobenzene (**2**‐Br) was used as a control system that cannot bind copper.

### 2.3 Coupling of 2 to gold electrodes by reduction of in situ generated 4‐bromobenzene‐diazonium

Several aryldiazonium compounds have been used to immobilize benzylic molecules at gold^[17]^ and glassy carbon electrodes.[^14^, ^17b^, ^18^] In the group of Bèlanger, it was discovered that a glassy carbon electrode grafted with **2** contains 0.38 nmol cm^−2^ using X‐ray photoelectron spectroscopy (XPS).^[18]^ In the cyclic voltammogram of reduction of **2**‐N_2_^+^ at glassy carbon, two peaks are observed. The first reduction peak is observed at 0.71 V *versus* RHE.^[17b]^ The nature of this peak is at present not fully understood. By scanning the potential up to this peak, partial blocking of the surface is observed in K_3_[Fe(CN)_6_] blocking experiments. This peak is thus already associated with the reduction of **2**‐N_2_^**+**^, but does not form a fully covered surface. The second reduction is associated with the reductive coupling of **2**‐N_2_^+^ and **2** ^•^ to the electrode surface and is observed at −0.09 V *versus* RHE. The peak is followed by a plateau reaching the vertex potential at −0.79 V *versus* RHE, which is also associated with the reduction of **2**‐N_2_^+^ and the **2** radical. In the second scan of the cyclic voltammogram, the reductive peak is no longer visible.

Gooding et al. immobilized **2** via the reduction of the in situ generated **2**‐N_2_^**+**^ at gold electrodes.^[17b]^ In the first scan of the cyclic voltammogram at a gold electrode, a reduction peak was reported at 0.36 V *versus* RHE. The peak is followed by a plateau. In the backward scan, the current drops to 0 A quickly. The reductive peak and the plateau are associated with the reduction of **2‐**N_2_^+^ at the gold electrode. In the second scan, the peak and plateau are no longer visible. The differences in the cyclic voltammetry between glassy carbon and gold electrodes indicate the coupling of organic molecules is dependent on the electrode material and consequently that it is impossible to directly compare the reduction of diazonium ions at different electrode materials.

In this study, the reductive coupling of **2** with the gold electrode was performed in a hanging meniscus configuration. In the first scan of the immobilization of **2**, a sharp reductive peak is observed at 0.34 V *versus* RHE. In the backward scan the current rapidly decreases to 0 A. The consecutive scan shows no reductive currents anymore, which indicates that the surface is fully covered with **2** after a single scan while scanning at 100 mV s^−1^. No oxidation processes are observed between 0.71 and −0.29 V *versus* RHE in all the scans, indicating that no oxidative degradation of the organic layer takes place. The results reported here are in good agreement with the electrochemistry of **2**‐NH_2_ reported by Gooding et al.^[17b]^ The formation of Au|**2** was further investigated using electrochemical quartz crystal microbalance (EQCM) techniques.

**Figure 2:**
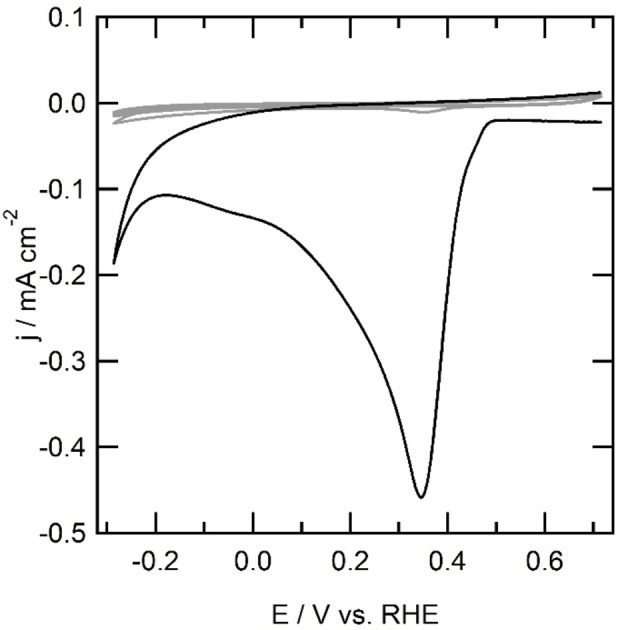
First scan (solid line) and second to fifth scan (grey lines) of the electrochemical immobilization of **2** at a gold electrode (0.05 cm^2^) at 100 mV s^−1^. Conditions: 1 mm **2**‐NH_2_, 1 mm NaNO_2_, 0.4 m HCl.

**Figure 3:**
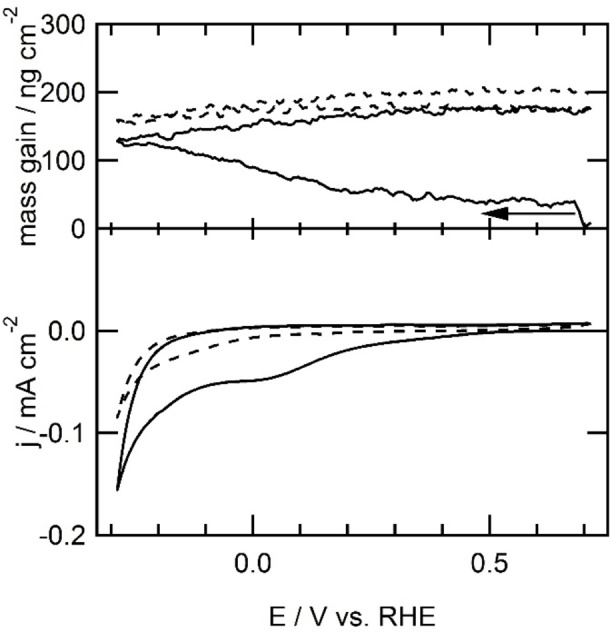
First scan (solid line) and second scan (dotted line) of electrochemical immobilization of **2** at a gold electrode (0.39 cm^2^) in combination with EQCM of bromobenzene by reduction of a 1 mm **2**‐N_2_^+^ ion solution generated in situ by addition of 4‐bromoaniline and sodium nitrite to form a 1 mm solution of both in 0.5 mHCl electrolyte solution at 100 mV s^−1^.

Given that the surface area of the EQCM electrode is larger (0.39 cm^2^ vs 0.05 cm^2^ real surface area, determined ex situ by measuring the charge transferred during the reduction of the gold oxide formed in 0.1 m HClO_4_ electrolyte solution^[19]^), and is not positioned in a hanging meniscus configuration, the voltammograms in these EQCM experiments show significantly broader features than discussed above. Using EQCM, the amount of **2** grafted onto the electrode surface was quantified. The reduction of the diazonium ion starts at 0.41 V *versus* RHE, as it did on the gold electrode in hanging meniscus configuration described above. A slow reduction process is observed over the whole forward scan below 0.41 V. The shape of the slow reduction of **2**‐N_2_^**+**^ shown in the cyclic voltammogram is different from the cyclic voltammogram in hanging meniscus configuration due to the differences in diffusion behavior (see Figures 2, 3 and S1). The total mass increase over the first scan is 145 ng cm^−2^, as is shown in the top panel of Figure 3, which corresponds to approximately 0.9 nmol cm^−2^.

The unit cell of gold FCC has a lattice constant of 406.5 pm.^[20]^ One gold atom in the Au(111) lattice therefore takes up 7.16×10^−16^ cm^2^. The total surface of the EQCM electrode thus contains approximately 5.45×10^14^ atoms. This indicates a coverage of approximately 40 molecules of **2** per 100 gold atoms. The total deposition of **2** measured by EQCM at a gold electrode (0.9 nmol cm^−2^) is higher than the coverage found in the group of Bèlanger on glassy carbon electrodes measured ex situ by XPS (0.38 nmol cm^−2^).^[18]^ During the first cycle of the cyclic voltammogram, 371 μC cm^−2^ reductive current has been recorded. This means that the faradaic efficiency of the formation of an Au‐**2** bond by reduction of **2**‐N_2_^**+**^ is approximately 48 % (see SI for details).

In order to assign potential ORR activity to copper sites that are bound to immobilized phenanthroline, we must rule out that catalytic reactions may occur at large areas of bare gold, or through unrestricted electron transfer from the electrode to oxygen in solution. We therefore investigated the electron transfer capabilities of Au|**2** electrodes in presence of the redox probe K_3_[Fe(CN)_6_], which is exclusively oxidized and reduced via outer sphere electron transfer via electron tunneling mechanisms. Using this approach, Gooding et al.^[17b]^ showed that that electron transfer from a phenyl bromide coated glassy carbon electrode to K_3_[Fe(CN)_6_] via outer sphere electron transfer is completely blocked.^[17b]^

**Figure 4:**
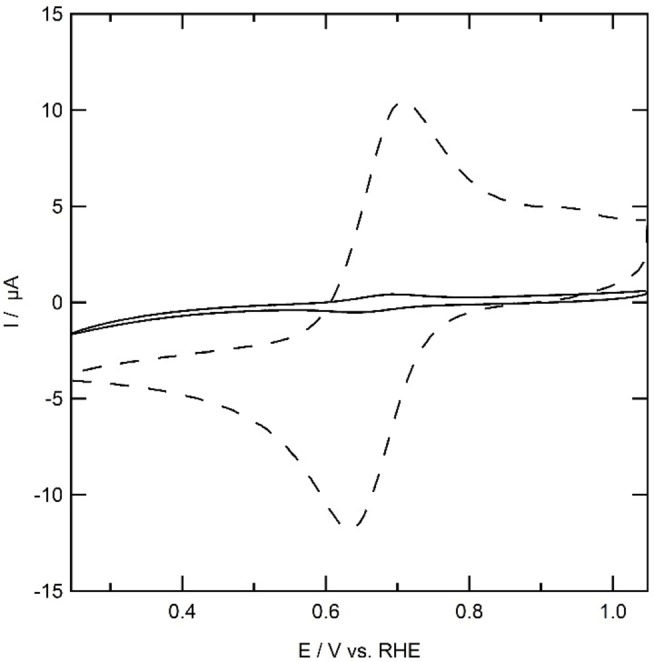
Outer sphere electron transfer at a gold electrode (dashed line) and a gold electrode modified with **2** using 1 mm K_3_[Fe(CN)_6_] in 0.1 m phosphate buffer and 0.05 m NaCl at pH 7.

If the electron transfer kinetics becomes slower due to the presence of a grafted non‐conductive layer, an increase in ΔE is expected together with lower peak currents, while E_1/2_ should remain unaffected. On a polished gold electrode, an Fe^II^/Fe^III^ redox couple is observed with E_1/2_=0.65 V (Figure 4). The peak of the oxidative wave is positioned at 0.29 V with a peak current of 9.2 μA and the reductive peak lies at 0.61 V with a peak current of −10 μA. If the surface is grafted with **2**, the current decreases twenty‐fold in a typical experiment while the peak potentials of the Fe^II^/Fe^III^ redox couple remain more or less at the same position, with E_p,o_=0.63 V and E_p,r_=0.28 V, resulting in E_1/2_=0.66 V. Since no apparent change in ΔE was observed, electron transfer must still occur at relatively fast rates. It therefore appears the surface of the gold electrode grafted with **2** is locally not completely covered. Since the surface exposed to the electrolyte might differ slightly each time a hanging meniscus is made, the small redox couple observed after grafting is attributed to redox activity at parts of the gold electrode that was not exposed to the grafting electrolyte in the immobilization experiment.

### 2.4 Reductive coupling of in situ generated 5‐diazo‐1,10‐phenanthroline to gold electrodes

The immobilization of **1** on glassy carbon electrodes was previously described by the group of Ekinci.^[14]^ The immobilization was performed in 46 % HBF_4_ between −0.1 and −1.2 V *versus* Ag/AgCl (approximately 0.1 to −1.0 V *versus* RHE) at −4 °C at 200 mV s^−1^, while sodium nitrite was added dissolved in acetonitrile. The surface coverage was determined to be 0.68 nmol cm^−2^ by XPS. The amount of **1** that is actually available for coordination was determined in the presence of [RuCl_3_]. By hanging the grafted electrode in a [RuCl_3_] solution and investigating the redox chemistry of the electrode afterwards, the number of available sites on the surface was determined to be 0.36 nmol cm^−2^.^[15]^

Several other methods to immobilize phenanthroline onto gold electrodes have been reported as well.[^16^, ^21^] In the group of Bertotti, 5‐bromo‐1,10‐phenanthroline was reduced in DMF to form the radical on the 5‐ position on the phenanthroline ring either stepwise via 5‐bromo‐1,10‐phenanthroline reduction to form a radical anion of 5‐bromo‐1,10‐phenanthroline. Formation of this radical anion was followed by loss of Br^−^ leaving a radical at the position where the bromine left. At this position, phenanthroline, in turn could couple to the Au working electrode.^[16]^ This forms a layer of Au|**1** on the electrode surface that in structure is identical to the reduction of the in situ generated **1**‐NH_2_. The thickness of the grafted layer was determined to be 125 nm using AFM. This indicates the formation of multiple layers of organic material on the surface of the electrode. Direct reductive coupling of 1,10‐phenanthroline to glassy carbon was investigated in the group of Bèlanger.[^15^, ^21^] Reduction of 1,10‐phenanthroline yields a coupling at the 4‐position. On the other hand, the reduction of in situ generated **1**‐N_2_^**+**^ couples exclusively at the 5‐position, since the radical formed on the phenanthroline molecule is situated in an sp‐orbital and thus cannot migrate over the benzylic rings via its π‐clouds. This implies that a different surface structure is obtained in case of **1**‐N_2_^**+**^ reduction compared to reduction of 1,10‐phenanthroline.

**Figure 5:**
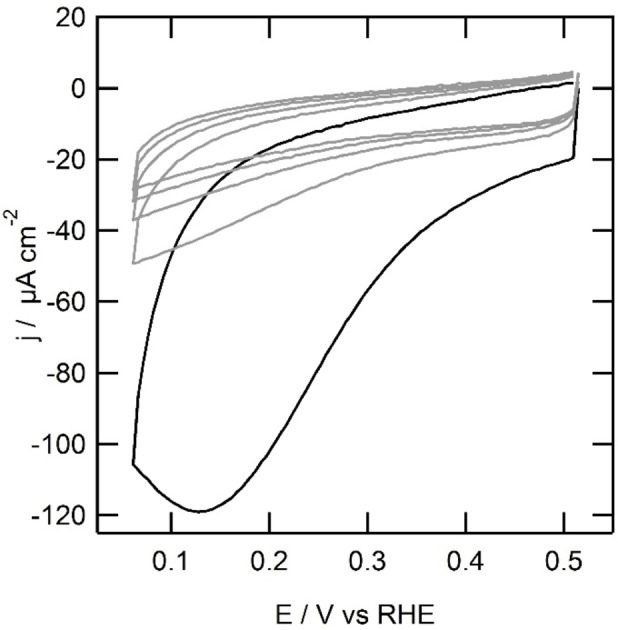
Immobilization of 1,10‐phenanthroline on a gold electrode (0.05 cm^2^) in hanging meniscus configuration from 1 mm **1**‐N_2_^+^ ions formed in situ by addition of sodium nitrite and **1**‐NH_2_ to form a 1 mm solution of both to a 0.5 m HCl electrolyte solution at 50 mV s^−1^ (first scan black, following scans in grey tone).

The reductive immobilization of **1** on gold was investigated using cyclic voltammetry in 0.5 m HCl solution (Figure 5). In contrast to the immobilization of **2**, the potential window was changed to 0.06 and 0.51 V, due to an oxidation observed above 0.51 V *versus* RHE. This oxidation is attributed to the formation of a ketone moiety on the 6‐position.^[22]^ At the start of the cyclic voltammetry at 0.51 V, a reductive current is observed. The negative current increases as a more reducing potential is applied. At 0.11 V *versus* RHE, a peak is observed. In the backward scan, the current decreases rapidly. In each following scan, a decrease in current is observed at the vertex potential of 0.06 V. In the fifth and last scan, significant current can still be observed, suggesting **1**‐N_2_^**+**^ is still being reduced in the fifth scan. It turned out that precise control over these radical reactions in combination with the surface morphology of the electrodes is difficult to control. Between different immobilization experiments of **1** under the same conditions, minor differences are observed with respect to the current and the peak position.

**Figure 6:**
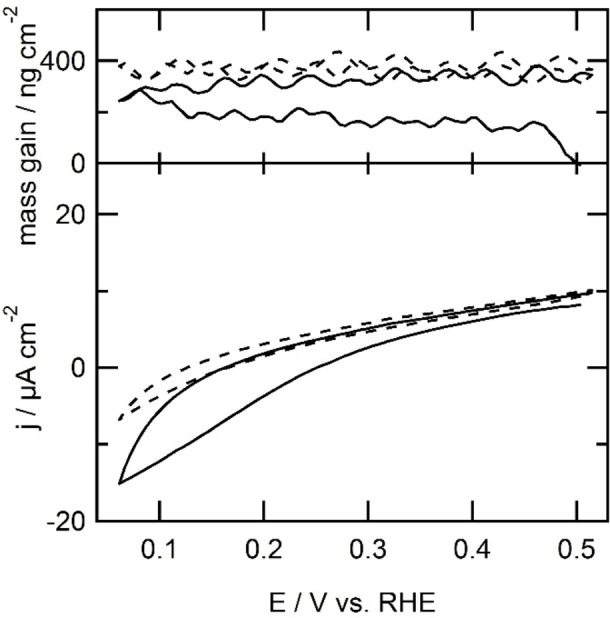
First scan (solid line) and second scan (dashed line) of electrochemical immobilization of **2** at a gold electrode (0.39 cm^2^) in combination with EQCM of bromobenzene by reduction of a 1 mm **2**‐N_2_^+^ ion solution generated in situ by addition of 4‐bromoaniline and sodium nitrite to form a 1 mm solution of both in 0.5 m HCl electrolyte solution at 100 mV s^−1^.

The amount of **1** that was immobilized on the gold electrode was quantified using EQCM (Figure 6). The mass of the electrode increases between 0.42 and 0.27 V *versus* RHE. This mass increase continues in the backward scan until 0.42 V. In the second scan, the mass remains stable, indicating that no further modification of the electrode takes place. Most likely an outer sphere electron transfer that reduces **1** takes place, which results in dimerization of **1** in the electrolyte solution. The reductive coupling of **1** at the EQCM electrode and the associated mass changes are displayed in Figure 6. At the start of the experiment at 0.5 V, an oxidative current is observed. Initially, the apparent mass of the electrode increases between the vertex potential of 0.51 V and 0.46 V. This increase in mass is most likely not associated with the reductive addition of **1** to the electrode surface. After all, a positive current is still being measured under these conditions, which is at present not understood. We therefore do not take into account the initial mass increase between 0.51 and 0.46 V. In the part of the voltammogram where reductive current is observed, the mass of the electrode increased by 77 ng cm^−2^. This corresponds to 0.4 nmol cm^−2^ or 18 molecules of **1** per 100 gold atoms.

**Figure 7:**
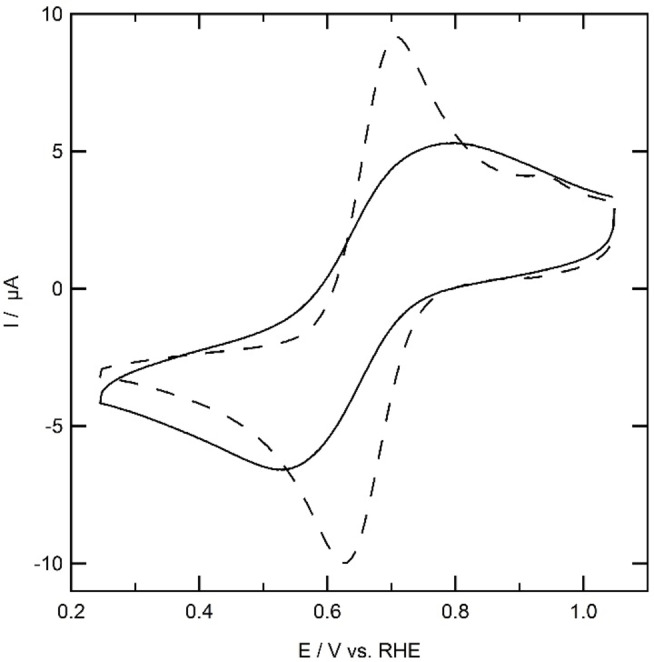
Outer sphere electron transfer at a gold electrode (dotted line) and a gold electrode modified with **1** using 1 mm K_3_[Fe(CN)_6_] in 0.1 m phosphate buffer and 0.05 m NaCl at pH 7.

K_3_[Fe(CN)_6_] was used as a redox probe to investigate the surface blocking properties of **1** attached to the gold working electrode (Figure 7). Both redox peaks of K_3_[Fe(CN)_6_] are significantly broader in presence of Au|**1** with respect to the redox probe at a bare gold electrode, with E_p,o_=0.78 V and E_p,r_=0.51 V. The halfway potential E_1/2_ is 0.65 V, which, as expected, is similar to the value found for the bare gold electrode. The peak current at the Au|**1** system drops to 5 μA, which is approximately half the peak current observed at the bare gold electrode. This drop in peak current and increased peak separation indicates that the outer sphere electron transfer rates from the electrode to the solution are retarded.

The current observed in the outer sphere redox behavior of **1** on gold differs from the immobilization of **1** on GC reported in the group of Ekinci.^[14]^ They observed a large decrease in current and do not report discernible redox peaks of the K_3_[Fe(CN)_6_] redox couple, but rather a positive and negative plateau. The higher surface blocking of the electrode observed by Ekinci et al. correlates well with the higher surface coverage that was observed in their experiments on glassy carbon.

Outer‐sphere electron transfer is significantly faster in case of Au|**1** (Figure 7) compared to Au|**2** (Figure 4). Considering that the diazocoupling reaction is likely irreversible and that moving from **1** and **2** over the gold surface is difficult, one may expect the smaller and more symmetric **1** to form a more densely packed monolayer on gold than **2**.

### 2.5 Oxygen reduction activity of modified gold electrodes

In oxygen reduction experiments at immobilized catalysts it is crucial to rule out any catalytic activity due to uncovered electrode material. The activity of an unmodified gold electrode was investigated between 0.63 and 0.23 V *versus* RHE in 0.1 m phosphate buffer and 0.05 m NaCl acidified to pH 4 using HCl. The onset potential for oxygen reduction is determined to be roughly 0.39 V, after which a catalytic wave is observed reaching a mediocre current of −410 μA cm^−2^ at the lower vertex potential of 0.23 V. Apparently the oxygen reduction activity of gold is heavily hampered by the presence of either chloride or phosphate.

**Figure 8:**
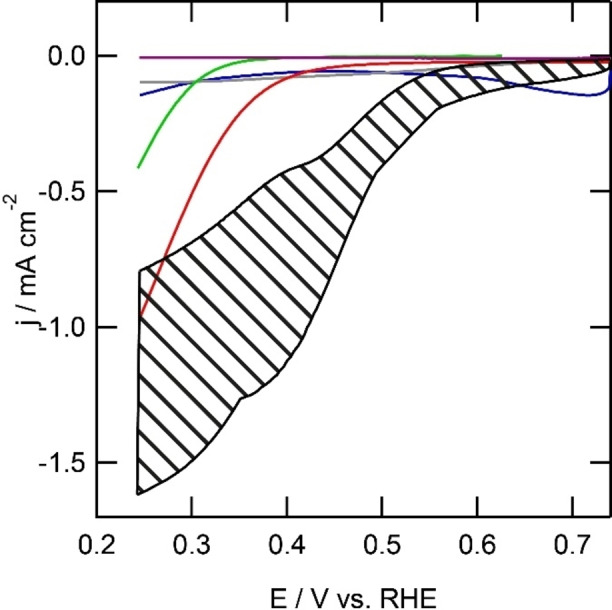
Electrochemical behavior of a bare gold electrode (green line), Au|**2**|Cu (purple), Au|Cu (blue line), Au|**1** (red line) and Au|**1**|Cu (black area) in presence of oxygen and Au|**1**|Cu in absence of copper (grey line) in 0.1 m phosphate buffer with 0.05 m NaCl acidified to pH 4 with HCl at 100 mV s^−1^. The black striped area represents the spread of current observed over different oxygen reduction experiments.

Another important potential catalytic species that must be ruled out is deposition of copper that forms under reductive potentials on the gold electrode.^[23]^ Oxygen reduction at a gold electrode in presence of 1 mM Cu^2+^ was investigated using cyclic voltammetry between 0.25 and 0.75 V. In the cyclic voltammogram, a reductive current of −6 μA is observed at the vertex potential of 0.75 V *versus* RHE (Figure 8). Two reversible redox events are observed in the cyclic voltammogram of the Au|Cu^II^ system in presence of oxygen. The first redox couple has a reductive peak at 0.72 V and an oxidative peak above the vertex potential of 0.75 V. The second redox event has a reductive shoulder around 0.55 V, which underlies the other reductive peak and an oxidative peak at 0.72 V. These peaks are associated with copper deposition on the gold working electrode. Below 0.45 V, a catalytic wave is observed with a maximum current of −145 μA cm^−2^ at the vertex potential. Apparently, oxygen reduction is suppressed by adding Cu^2+^ to the electrolyte solution. The current at 0.25 V in absence of Cu^2+^ is approximately three times higher than the current at a gold electrode in presence of Cu^2+^.

At an electrode modified with **2** no outer sphere electron transfer with K_3_[Fe(CN)_6_] is observed. Since it is unlikely for **2** to bind copper ions from the solution, it is not expected that Au|**2**|Cu^II^ will show any oxygen reduction activity. In the single sweep voltammetry of Au|**2**|Cu^II^ in presence of oxygen, no current is observed. This shows that it is therefore possible to block oxygen reduction by grafting the electrode surface with a layer of organic molecules, even in the presence of dissolved copper species. Since no outer sphere electron transfer was observed with Au|**2**|Cu^II^, two reasons for the absence of oxygen reduction could be given. Firstly, there is the absence of electron transfer, meaning it is impossible to get electrons from the electrode to the surface via the grafted layer. Secondly, Au|**2** cannot coordinate to copper, indicating no surface immobilized complex can be formed to reduce oxygen.

In absence of copper, the electrode modified with **1** was investigated for the oxygen reduction reaction. In the single sweep voltammogram, reductive current is observed with an onset potential of 0.45 V *versus* RHE. A catalytic wave with a maximum activity of 970 μA cm^−2^ was observed. The current is 2.5 times higher than the oxygen reduction activity observed on an unmodified electrode in absence of copper (−970 μA cm^−2^ for Au|**1** *versus* −410 μA cm^−2^ for a bare gold electrode). With **1** immobilized on a gold electrode, the onset potential for the reductive current is shifted positively with 100 mV compared to an unmodified gold electrode in presence or absence of Cu^II^.

An oxygen free solution of Au|**1**|Cu^II^ does not show any redox couples. Oxygen reduction at Au|**1**|Cu^II^ was observed with an onset potential that varied between 0.40 and 0.35 V *versus* RHE within different experiments. The activity at the lower vertex potential ranged between −0.80 to −1.6 mA cm^−2^ over different experiments. The onset shifted positively by 150 to 200 mV compared to the unmodified gold electrode in presence or absence of copper ions in solution, while the onset shifted 100 to 150 mV compared to Au|**1**. The activity is two to four times larger than the unmodified gold electrode in absence of Cu^II^ and equal to two times larger than Au|**1** (Figure 8).

As mentioned above, and in line with the immobilization experiments, the oxygen reduction activity for Au|**1**|Cu^II^ was not exactly the same over all experiments. In some experiments a minor shoulder was observed between 0.50 and 0.30 V. The maximum current observed at the vertex potential of 0.25 V differed from −0.80 to −1.6 mA cm^−2^ in different experiments. The overpotential for oxygen reduction ranged from 0.83 to 0.88 V. The differences in activity and onset potential may be due to the different surface structures formed by the reduction of **1**‐N_2_^**+**^. A different explanation may be the number of defects or the surface roughness, which would increase the number of **1** molecules adsorbed on the electrode surface. Nevertheless, in all experiments, the activity of the Au|**1**|Cu^II^ system surpassed the activity observed on a bare gold electrode.

**Figure 9:**
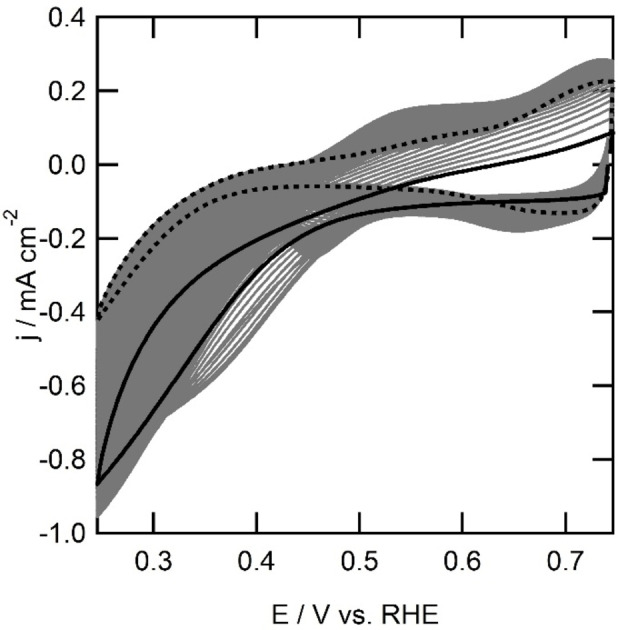
Electrochemical behavior of a gold electrode (0.05 cm^2^ geometric surface area) grafted with **1** in presence of oxygen in 0.1 m phosphate buffer with 0.05 m NaCl acidified to pH 4 with HCl at 100 mV s^−1^. First scan is depicted in black, 200^th^ scan is depicted in dotted black line.

In order to investigate the stability of the 1,10‐phenanthroline immobilized system, cyclic voltammetry over 200 cycles was performed (Figure 9). A decrease in current is observed from 960 μA cm^−2^ in the first scan to 400 μA cm^−2^ in the 200^th^ scan. Accompanied by the decrease in activity is the appearance of two oxidation and reduction peaks. There is an oxidation peak increasing with each scan at 0.55 V which is accompanied by a reduction at 0.47 V. The second redox couple has an oxidative peak at 0.73 and a reduction at 0.68 V.

Apparently, the Au|**1**|Cu^II^ system is a very dynamic system. The decrease in catalytic activity suggests that deactivation of the catalyst or degradation of the organic layer takes place. The Au‐**1** bond could be broken and the complex may leach into the electrolyte solution. This would indicate that the gold surface becomes exposed to the electrolyte and that copper ions from the solution can adsorb onto the gold electrode. This would result in redox activity similar to the Au|Cu^II^ system. However, the redox couples do not completely match between Au|Cu^II^ and Au|**1**|Cu^II^ after 200 cycles of oxygen reduction. Another explanation could be the formation of thick copper layers on top of the phenanthroline layer. The composition of Au|**1**|Cu^II^ as a function of scan number and thus time was therefore investigated by XPS (Figures S11 to S14).

### 2.6 XPS analysis of Au|1 after different stages of oxygen reduction

**Figure 10:**
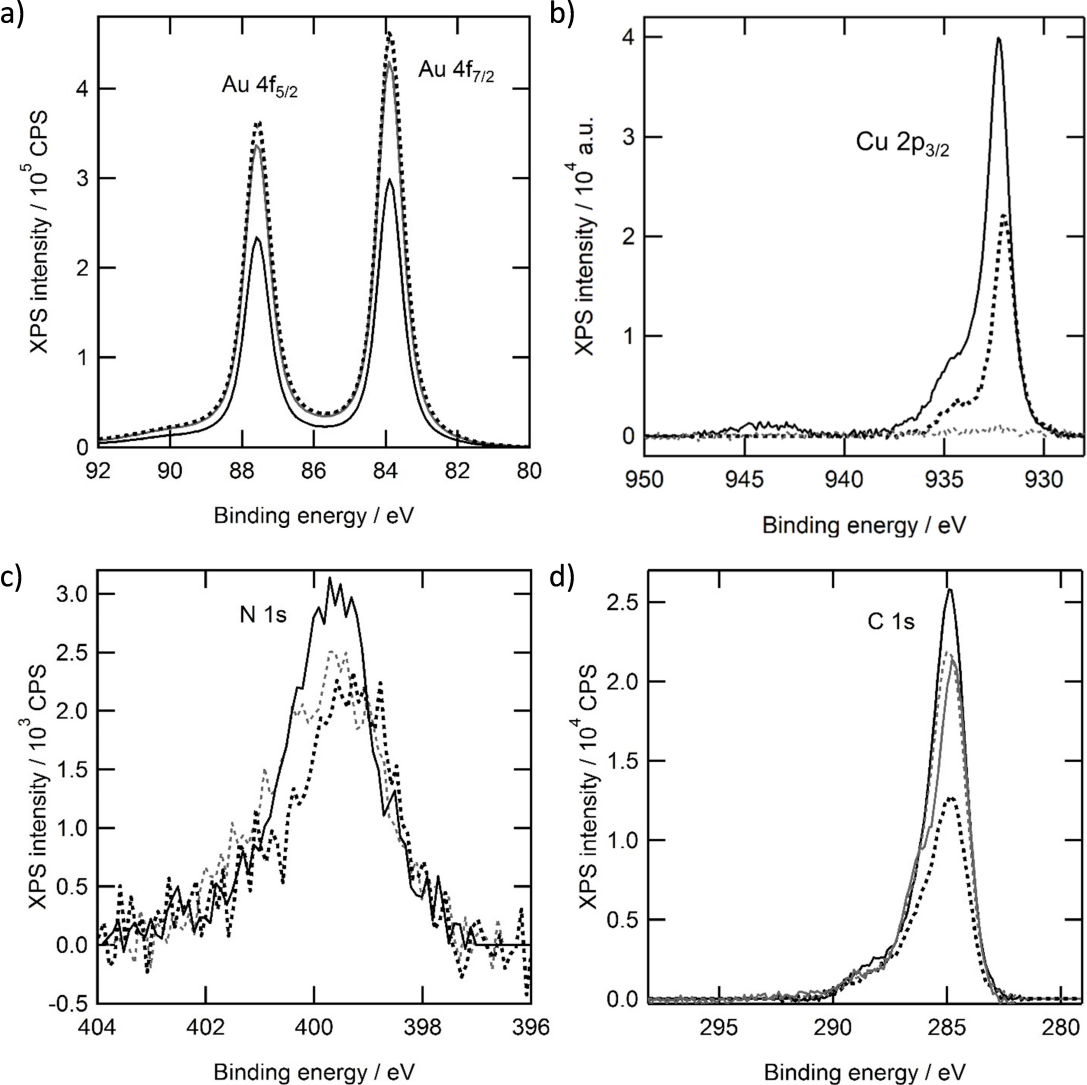
Ex situ X‐ray photoelectron spectra of gold electrodes in the Au 4 f region (a), Cu 2p_3/2_ region (b), N 1s region (c) and C 1s region (d) of an unmodified electrode (gray solid line), an electrode grafted with **1** (gray dotted line), after 25 cycles of oxygen reduction (black solid line) and after 200 cycles of oxygen reduction (black dotted line).

The Au 4f_7/2_ peaks of the unmodified gold electrode are observed at 83.9 eV, which corresponds to literature values of 83.8 eV and 83.9 eV (see Figure 10a).^[24]^ In the Au|**1** system, the binding energy and the total intensity of the Au 4f_7/2_ peak does not change, indicating that the adsorbed layer of **1** is very thin. After 25 cycles of oxygen reduction in the Au|**1**|Cu^II^ system, a difference is observed and the intensity of the Au signals dropped by approximately 30 %. Upon 200 cycles of oxygen reduction the intensity of the Au 4 f peaks return to the original value of the unmodified electrode. Apparently a relatively thick layer of copper builds up on the electrode surface of Au|**1** and disappears over time. The position of the gold peaks does not change during oxygen reduction or by immobilization of **1** on the electrode, as one would expect.

The copper layer that is formed during the oxygen reduction reaction is accompanied by the appearance of the Cu 2p_3/2_ peaks at 932.3 eV after 25 cycles of voltammetry (Figure 10b). The position of the Cu 2p_3/2_ peaks is in the same region where these peaks are observed for metallic copper and Cu(I) oxide. Values between 931.5 and 932.4 eV for Cu(0) and between 931.6 and 932.0 eV for Cu_2_O have been reported in the literature.^[25]^ It is very difficult to assign the precise oxidation state of copper based on its binding energy, especially since little XPS data is available for molecular copper systems.^[26]^ Also weak satellites are observed between 940 and 947 eV, which are indicative of the presence of some Cu(II) as well.^[27]^ In this discussion it is important to note that a deposit of a reduced Cu species forms on the electrode; we estimate that Cu^0^ is most likely formed under catalytic oxidation and that oxidation to the +I oxidation state may occur due to post catalysis air exposure and handling. After 200 cycles of oxygen reduction, the total intensity of copper decreases to approximately 50 % of the value observed after 25 cycles of oxygen reduction. The Cu 2p_3/2_ peak position shifts to a lower binding energy of 932.0 eV and the satellites between 940 and 947 eV disappear, indicating a further reduction of the copper species on the surface. The decrease in intensity for the Cu 2p peaks, and thus the formation of a thinner layer on top of the gold electrode is reflected by the higher intensity observed for the Au 4f peaks after 200 cycles, which returned to values similar to bare gold and Au|**1**.

The N 1s signals of the grafted electrode are observed at 399.6 eV (Figure 10c). Upon performing 25 cycles of oxygen reduction, the peak position and intensity do not change significantly compared to the electrode prior to the catalytic reaction. After 200 cycles of oxygen reduction the binding energy shifts to a slightly lower binding energy of 399.4 eV, suggesting either a structural change of the organic layer, or an indirect effect as the result of further reduction of the ligated copper sites.

### 2.7 Mechanistic implications

In a previous study Chidsey et al. have immobilized phenanthroline copper systems on glassy carbon electrodes by employing flexible triazole linkers and proposed oxygen reduction to occur via a binuclear pathway wherein the reduction of oxygen would predominantly occur.^[28]^ The rigidity of our phenanthroline system would rule out such bimolecular pathways. Also, formation of Cu(phen)_2_ species does not seem very likely to occur upon addition of Cu to Au|**1**. The catalytic activity of Au|**1**|Cu^II^ is superior to that of gold and copper deposited on gold under the chosen catalytic conditions, which suggests that the catalytic activity must be related to the presence of both copper and phenanthroline. However, the obtained XPS data rule out that a well‐defined Cu^II^(phen) species is obtained as was identified by EPR in solution upon mixing of Cu(OTf)_2_ in NaCl with one equivalent of phenanthroline. Instead, a thicker layer of copper seems to be formed on the electrode surface. In a previous study we have shown that a Cu(I) species forms on the electrode surface of SAM‐functionalized Cu(tmpa), wherein the copper to ligand ration has shifted significantly towards higher copper loadings.^[26]^ This species was shown to be inactive in the oxygen reduction reaction, though. In contrast, the copper layer attached to Au|**1** appears to be significantly thicker on basis of the disappearance and reappearance of the intensity of the gold XPS signal. Simultaneously, the catalytic activity appears to be directly correlated with the copper content.

Molecular copper systems are typically highly dynamic under electrochemical conditions, which probably relates to the fast ligand exchange kinetics of copper(II) species and to facile formation of copper deposits.^[8]^ The Au|**1**|Cu^II^ reported here appears to be more stable over time, but after 100 voltammetry scans, the copper content of Au|**1**|Cu^II^ diminishes and the catalytic activity decreases. These phenomena coincide with a slight shift in binding energy of the nitrogen atoms that are still present in the catalytic sample.

## 3 Conclusion

The immobilization of phenanthroline via a diazonium coupling reaction leads to a well‐defined thin organic layer on gold which allows for binding of copper ions from the solution. However, electrochemical reduction in the presence of Cu^2+^ does not only lead to the binding of copper to phenanthroline, but also results in a relatively thick layer of copper that does not relate to molecular Cu(phen) species. Nevertheless, this copper layer is active for the oxygen reduction reaction and the activity depends on the presence of both copper and the organic ligand.

## Experimental Section

### Reagents and materials

1,10‐phenanthroline (Sigma Aldrich, 97 %), 2–2’‐bipyridine (Alfa Aesar, 99 %), 4,7‐dimethyl‐1,10‐phenanthroline (Sigma Aldrich), 1,10‐phenanthroline‐5‐amine (Sigma Aldrich, 97 %), 4‐bromoaniline (Sigma Aldrich, 99 %), NaNO_2_ (Merck, 99.9 %), K_3_Fe(CN)_6_ (Sigma Aldrich, 99.98 %) and Cu(OTf)_2_ (Alfa Aesar, ≥99 %) were used as received. Electrolyte solutions were prepared with HClO_4_ (Merck suprapur, 70 %), HCl (Merck, 37 %), Na_2_HPO_4_ (Merck, 99.9 %), NaH_2_PO_4_ (Merck, 99.9 %), NaCl (Merck, 99.9 %) and were prepared with MilliQ water (>18.2 MΩ cm resistivity). Argon and oxygen (5.0) were purchased from Linde Gas.

### EPR spectroscopy

The EPR experiments were performed on a Bruker EMXplus X‐band. Simulation of the spectra was performed using the W95EPR software. The spectra were simulated using the W95EPR software. The EPR samples were prepared by dissolving different concentrations, ranging from 1 mm to 0.1 mm, of 1,10‐phenanthroline together with 0.1 mm Cu^II^, 0.1 m phosphate buffer, 0.05 m NaCl acidified to pH 4 using HCl. The samples were recorded at 77 K.

### Electrochemical methods

All electrochemical experiments were performed on an Autolab PGSTAT 128 N with integrated EQCM module in one‐compartment 25 mL glass cells in three‐electrode setups. A gold working electrode (99.995 %, Alfa Aesar, 0.05 cm^2^ geometric surface area) was used in a hanging meniscus configuration. Platinum (99.99 %, Alfa Aesar) was used as a counter electrode and a Ag/AgCl (3 m KCl) purchased from Autolab was used as reference electrode.

The Au electrode was cleaned by oxidation at 10 V *versus* a graphite rod counter electrode in 10 % H_2_SO_4_ for 30 s. This was followed by a 6 M HCl bath for 20 s, followed by flame annealing. The electrode was then electrochemically polished by cycling between 0 and 1.75 V *versus* RHE (E_start_=0.7 V) at 1 V s^−1^ in 0.1 m HClO_4_. Modification of gold electrodes was performed in 0.5 M HCl electrolyte with 1 mm 1,10‐phenanthroline‐5‐amine (97 %, Sigma Aldrich) or 4‐bromoaniline (99 %, Sigma Aldrich). The solution was deaerated for 30 min with argon (Linde 5.0). After deaeration, 3.4 mg NaNO_2_ (puriss., Sigma Aldrich) was added to the electrolyte solution. After 60 seconds, argon was blanketed over the electrolyte solution and a cyclic voltammogram was started. For the 4‐bromoaniline, the Au electrode was cycled between −0.5 and 0.5 V *versus* Ag/AgCl at 100 mV s^−1^ for 5 cycles. For 1,10‐phenanthroline‐5‐amine, the Au was cycled −0.2 and 0.3 V *versus* Ag/AgCl at 50 mV s^−1^ for 10 cycles.

Oxygen reduction experiments were performed in 0.05 m NaH_2_PO_4_ (99.99 %, Merck), 0.05 m Na_2_HPO_4_ (99.99 %, Merck), 0.05 m NaCl (99.99 %, Merck) and 0.6 mm HCl (37 %, VWR International). In case of catalytic experiments, 1 mm Cu(OTf)_2_ was added to the electrolyte solution. Prior to oxygen reduction experiments, oxygen was bubbled through the electrolyte for at least 20 minutes, while during experiment, oxygen was blown over the solution. Apart from the EQCM experiments and the experiments wherein the XPS samples are created, all electrochemical experiments were performed in a hanging meniscus configuration. In a hanging meniscus configuration, the electrode is situated in the headspace‐electrolyte interface (left panel Figure S1). Prior to experiment, the electrode is not in contact with the electrolyte solution. The electrolyte containing 1 mm 1,10‐phenanthroline‐5‐amine or 4‐bromoaniline is deaerated by bubbling argon through the electrolyte solution. After deaeration sodium nitrite is added as a solid to the electrolyte, while argon was continued to bubble through the electrolyte solution to promote mixing. One minute after addition of sodium nitrite, argon is blown over the headspace and the working electrode is brought in contact with the electrolyte, making a hanging meniscus.

### EQCM setup

The electrochemical quartz crystal microbalance consisted of a PEEK cell purchased from Autolab. The cell was degassed with Ar (Linde, 5.0) prior to experiment. A gold working electrode (0.35 cm^2^ geometric surface area) on a quartz crystal was used as received. Platinum was used as counter electrode and the experiments were measured *versus* a Ag/AgCl (3 M KCl) reference electrode. In the EQCM setup, the electrode is situated in the bottom of the cell (see right panel of Figure S1). This contrasts the electrochemical experiments wherein a hanging meniscus configuration was used, where the electrode sits in the liquid‐headspace interface

Prior to the experiment, the oscillator of the EQCM needs to be stabilized by running it for 30 minutes. This is done in 3 mL MilliQ water. Simultaneously the 25 mL electrolyte solution is deaerated in separate flask by bubbling argon through the electrolyte solution. After stabilization of the EQCM oscillator, the following steps were taken in quick succession. First the NaNO_2_ was added to the electrolyte solution in the separate flask, while bubbling argon through the electrolyte solution to improve mixing. Secondly the EQCM oscillator was turned off and the MilliQ water was removed from the EQCM cell. Thirdly argon was blown into the side of the EQCM cell, so that it would form a blanket over the electrolyte solution once it is added. 3 mL of the electrolyte solution was inserted in the EQCM cell. The counter and reference electrode were then placed in the electrolyte solution and the starting potential was applied, after which the experiment was started immediately.

### XPS Analysis

The XPS measurements were carried out with a Thermo Scientific K‐Alpha, equipped with a monochromatic X‐ray source and a 180° double focusing hemispherical analyzer with a 128‐channel detector. Samples were shipped and handled in ambient air and conductively clamped on the XPS sample holder via the Au layer. Spectra were obtained using an aluminum anode (hv(Al Kα)=1486.6 eV) operating at 72 W and a spot size of 400 μm. Survey scans were measured at a constant pass energy of 200 eV and region scans at 50 eV. The background pressure was 2×10^−8^ mbar and during measurement 4×10^−7^ mbar argon because of charge compensation. Binding energy calibration of the spectra was performed by setting the Au 4 f peak to that of bare Au electrode (BE(Au 4f_7/2_=83.9 eV).

## Conflict of interest

The authors declare no conflict of interest.

## Supporting information

As a service to our authors and readers, this journal provides supporting information supplied by the authors. Such materials are peer reviewed and may be re‐organized for online delivery, but are not copy‐edited or typeset. Technical support issues arising from supporting information (other than missing files) should be addressed to the authors.

Supporting Information
