# Supplementary material for: A Heterogenized Copper Phenanthroline System to Catalyze the Oxygen Reduction Reaction
Source: ChemElectroChem. 2022 Feb 2;9(3):e202101365. doi: 10.1002/celc.202101365 (PMC9305121; doi:10.1002/celc.202101365)
Supplement: Supplementary file 1 — Supporting Information [file CELC-9-0-s001.pdf]

ChemElectroChem

Supporting Information

A Heterogenized Copper Phenanthroline System to Catalyze the Oxygen Reduction Reaction

Cornelis J. M. van der Ham, Damy N. H. Zwagerman, Longfei Wu, Jan P. Hofmann, and Dennis G. H. Hetterscheid*

EXPERIMENTAL SETUP

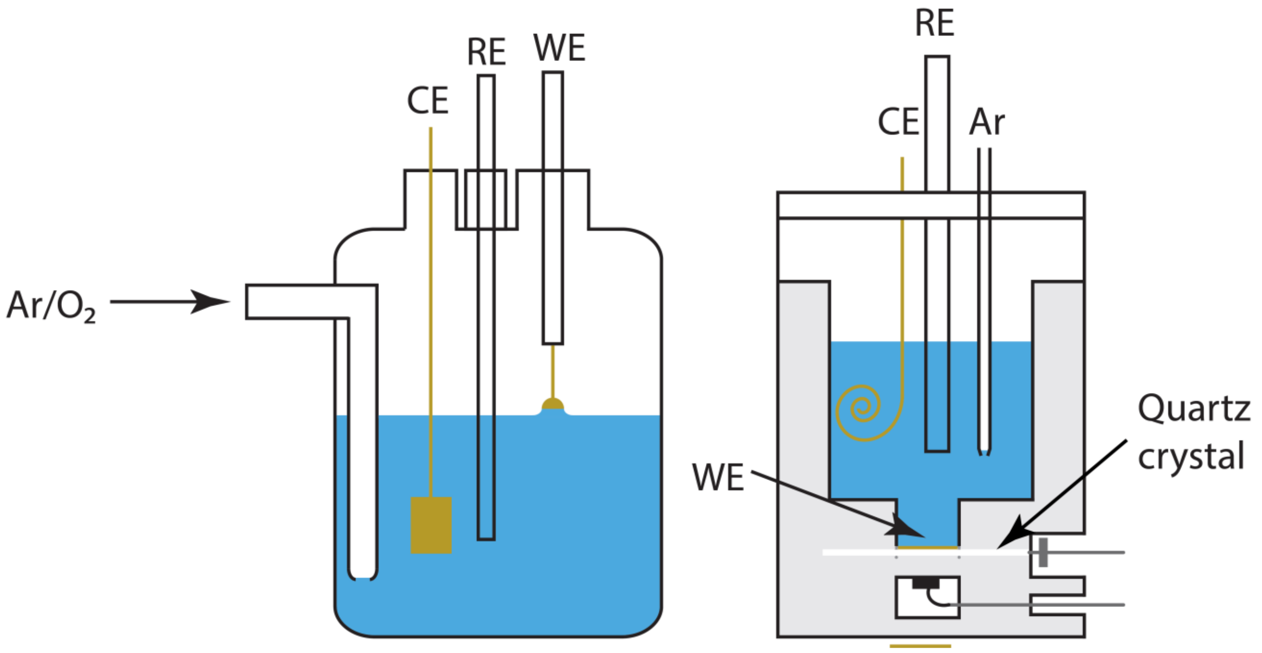

Figure S1: Schematic representations of electrochemical cells with an electrode used in hanging meniscus configuration (left) and the electrode sitting in the bottom of the EQCM cell (right).

ELECTROCHEMISTRY OF Cu(phen)

Copper phenanthroline solutions were formed *in situ* in by preparing an electrolyte solution with 1 mM Cu^{II} and 1 mM 1,10-phenanthroline and 1 mM Cu^{II} and 2 mM 1,10-phenanthroline in 0.1 M phosphate buffer containing 0.05 M NaCl acidified to pH 4 using HCl. Whereas the 1:2 mixture shows an apparent reversible redox couple, no well-defined redox couples were observed in an electrolyte solution containing 1 mM Cu^{II} and 1 mM 1,10-phenanthroline.

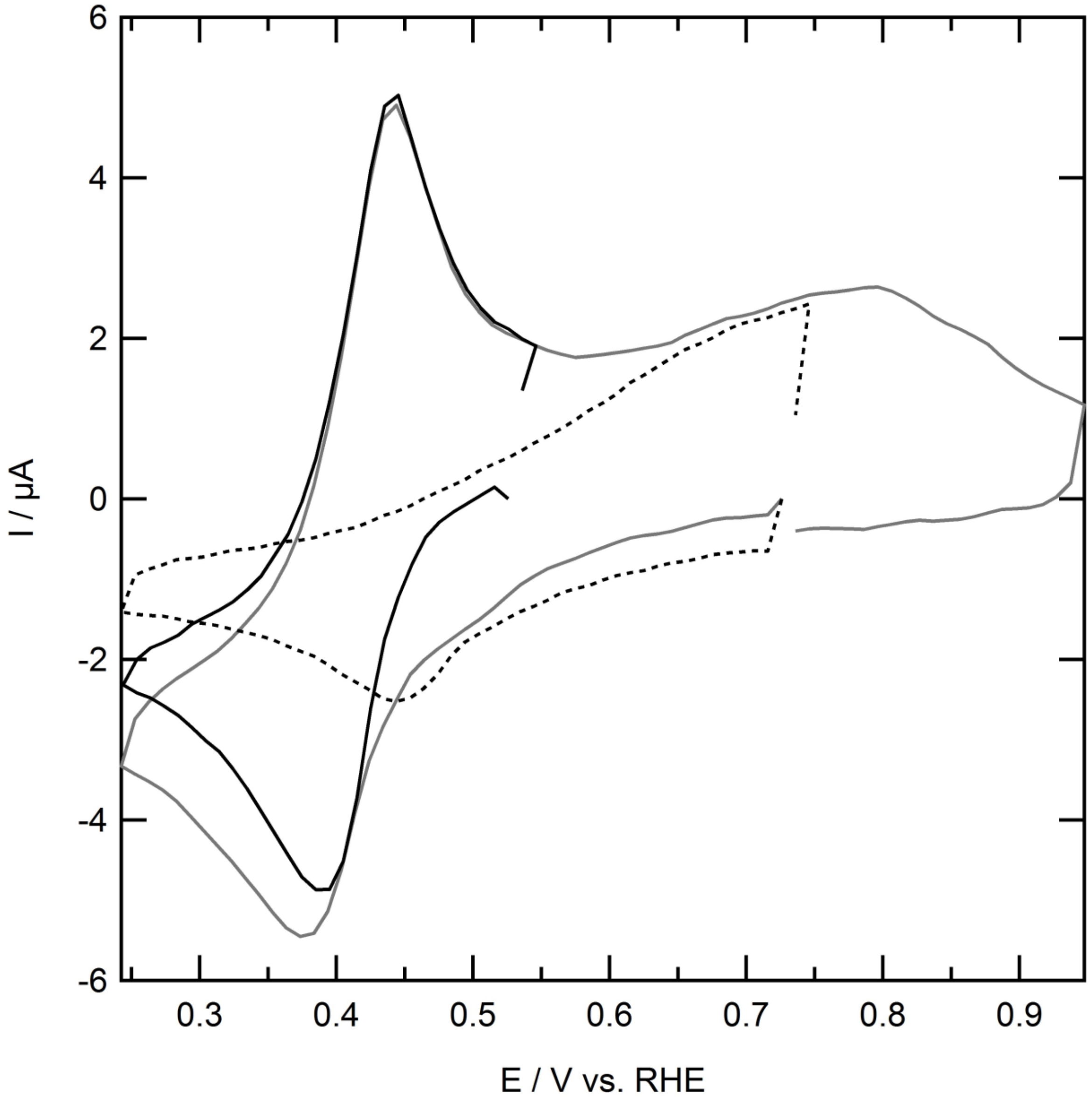

Figure S2: Cyclic voltammogram of a gold electrode under argon in a 1 mM solution of Cu^{II} and 2 mM 1,10-phenanthroline (solid lines) and in a 1 mM solution of Cu^{II} and 1 mM 1,10-phenanthroline (dotted line) in a 0.1 M phosphate buffer with 0.05 M NaCl acidified to pH 4 at 100 mV s^{-1}.

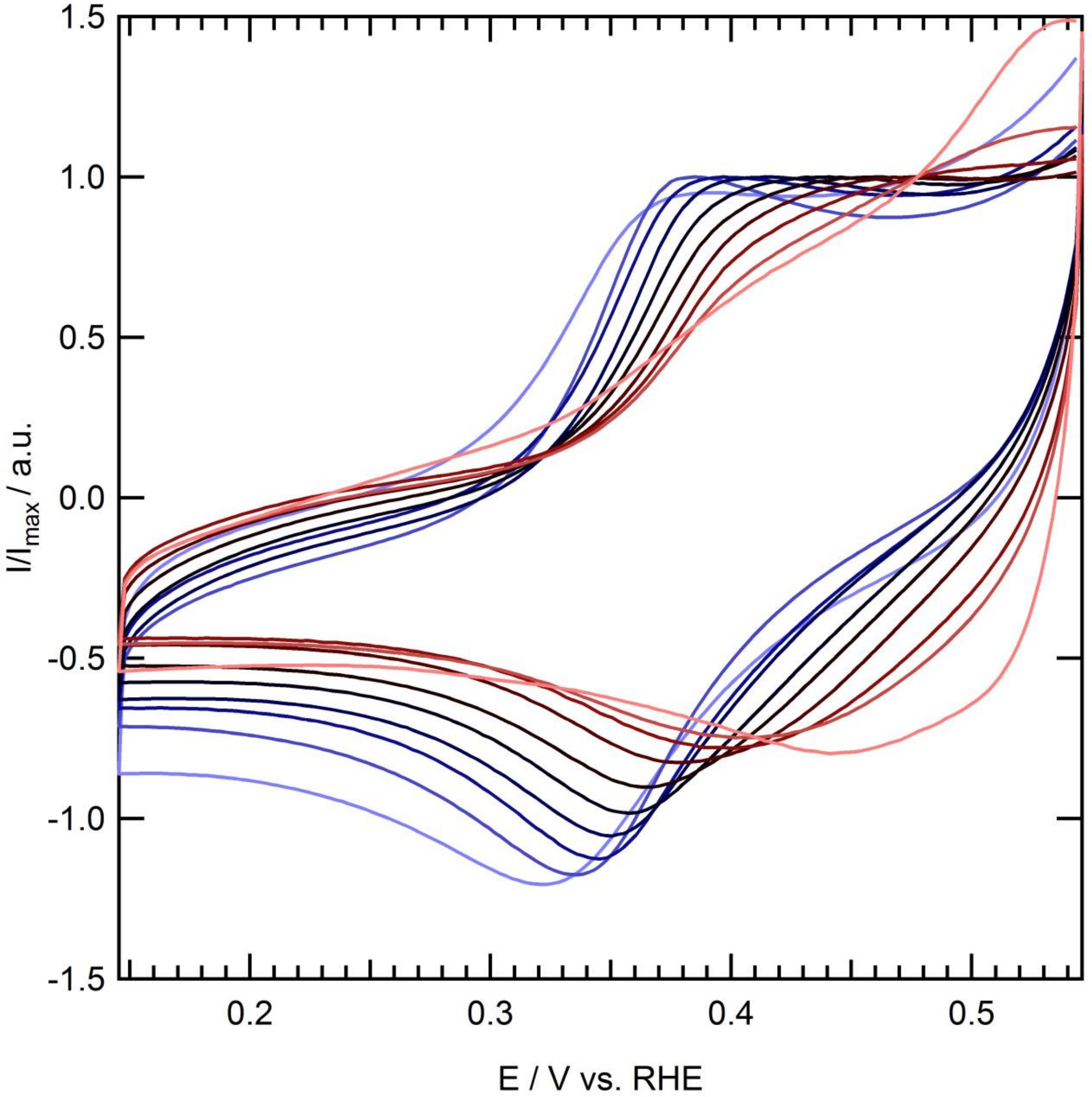

Figure S3: Cyclic voltammograms with a range of 1,10-phenanthroline concentration from 1 mM (blue line) to 0.1 mM (red line) and 0.1 mM of Cu_{II} in a 0.1 M phosphate buffer with 0.05 M NaCl acidified to pH 4 at 100 mV s^{-1}.

Similarly to Figure S2 but at a 0.1 mM concentration of copper and 1,10-phenanthroline (thus at a 1:1 ratio of 1,10-phenanthroline to Cu^{II}) no reversible redox behavior is observed whatsoever. The $E_{1/2}$ shifts from 0.41 V at a 2:1 ratio to 0.34 V at a 10:1 ratio of 1,10-phenanthroline to Cu^{II}. This indicates that the balance shifts from free Cu^{2+} and Cu^{II}(phen) to $Cu^{II}(phen)_2$ at a high concentration of 1,10-phenanthroline.

We also explored the closely related bipyridine (bipy) and dimethylphenanthrole (DMP) ligands, to verify whether a better optimum between catalyst stability catalyst activity could be found. The redox behavior of complexes with bipy and DMP ligands was investigated in a 1 to 10 copper to ligand ratio. Cyclic voltammetry was performed with an *in situ* generated complex from 1 mM bipy and 0.1 mM Cu^{II} in 0.1 M phosphate buffer with 0.05 M NaCl acidified to pH 4 using HCl. In the cyclic voltammogram two redox events are observed (Figure S4a). A reversible redox couple is visible at 0.39 V *verus* RHE with low peak separation. A low peak separation is indicative of a surface adsorbed species. Between 0.6 and 0.7 V, an oxidative peak is observed, which is accompanied by a reductive peak at 0.52 V *versus* RHE. At high scan rate, the oxidative peak between 0.6 and 0.7 V broadens and the oxidative wave of the reversible redox couple at 0.39 V is no longer visible.

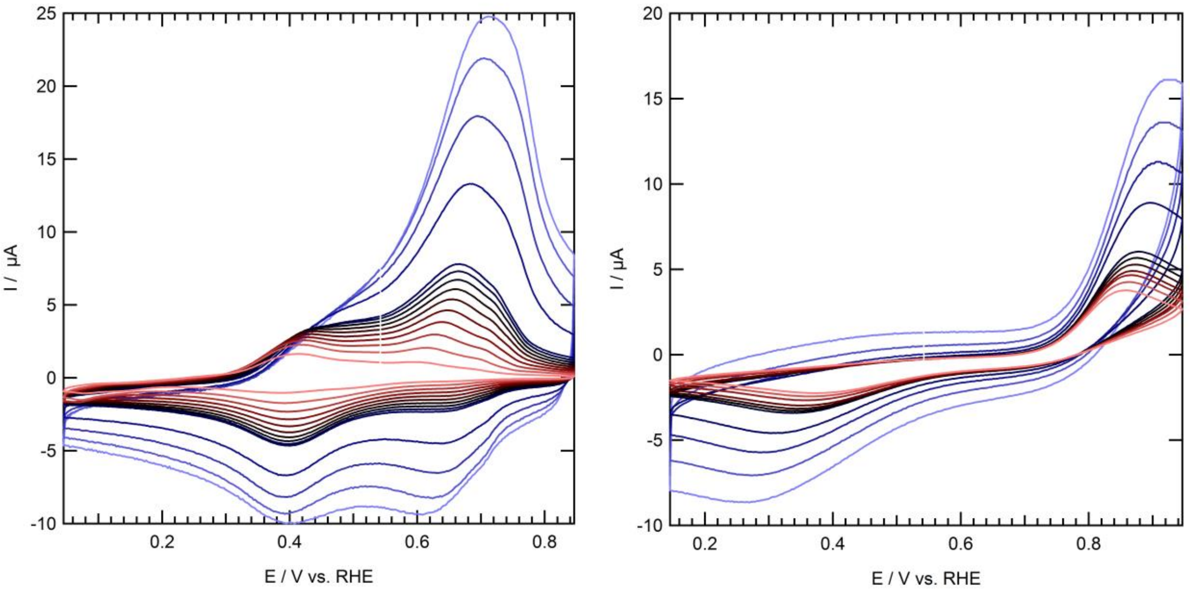

Figure S4: Cyclic voltammogram at scan rates from 10 (red line) to 500 (blue line) mV s^{-1} of a gold electrode with 1 mM solutions of a) 2,2'-bipyridine and b) 4,9-dimethyl-1,10-phenanthroline together with 0.1 mM Cu^{II}, 0.1 M phosphate buffer and 0.05 M NaCl acidified to pH 4 using HCl.

By calculating the peak current of either the reduction or the oxidation process of a reversible redox process, the homogeneity of a molecular process can be determined. For one-electron transfer processes in solution, a linear relationship between the peak current and the squire root of the scan rate is expected, according to the Randles–Ševcik relation (Equation S1), where i_p is the peak current, n is the number of electrons, F is the Faraday constant, v is the scan rate, A is the electrode area, D_0 is the diffusion constant, R is the gas constant and T is the temperature.

$$i_p = 0.466nFAC^0\left(\frac{nFvD_0}{RT}\right)^{\frac{1}{2}} \quad (S1)$$

For the Randles–Ševcik relation, the peak position of the redox couple needs to be constant with changing scan rate. The peak position of the redox couples of cyclic voltammograms of *in situ* generated complexes

from 1 mM 1,10-phenanthroline and 0.1 mM Cu^{II} stays the same while changing the scan rate from 10 to 500 mV s^{-1} with $E_{1/2}$ = 0.81 V *versus* RHE (Figure S5). At scan rates of 200 mV s^{-1} and higher (blue lines), the oxidative peak becomes less well-defined. Therefore, the reductive peak was used to investigate the homogeneity of the catalytic process.

The peak current of cyclic voltammograms with a 10:1 ratio of ligand to Cu^{II} is linear with the square root of the scan rate (Figure S5). Moreover, the trend line goes through the origin when it is extrapolated. This indicates the complex behaves as a dissolved molecular species which exhibits one-electron transfer to the working electrode.

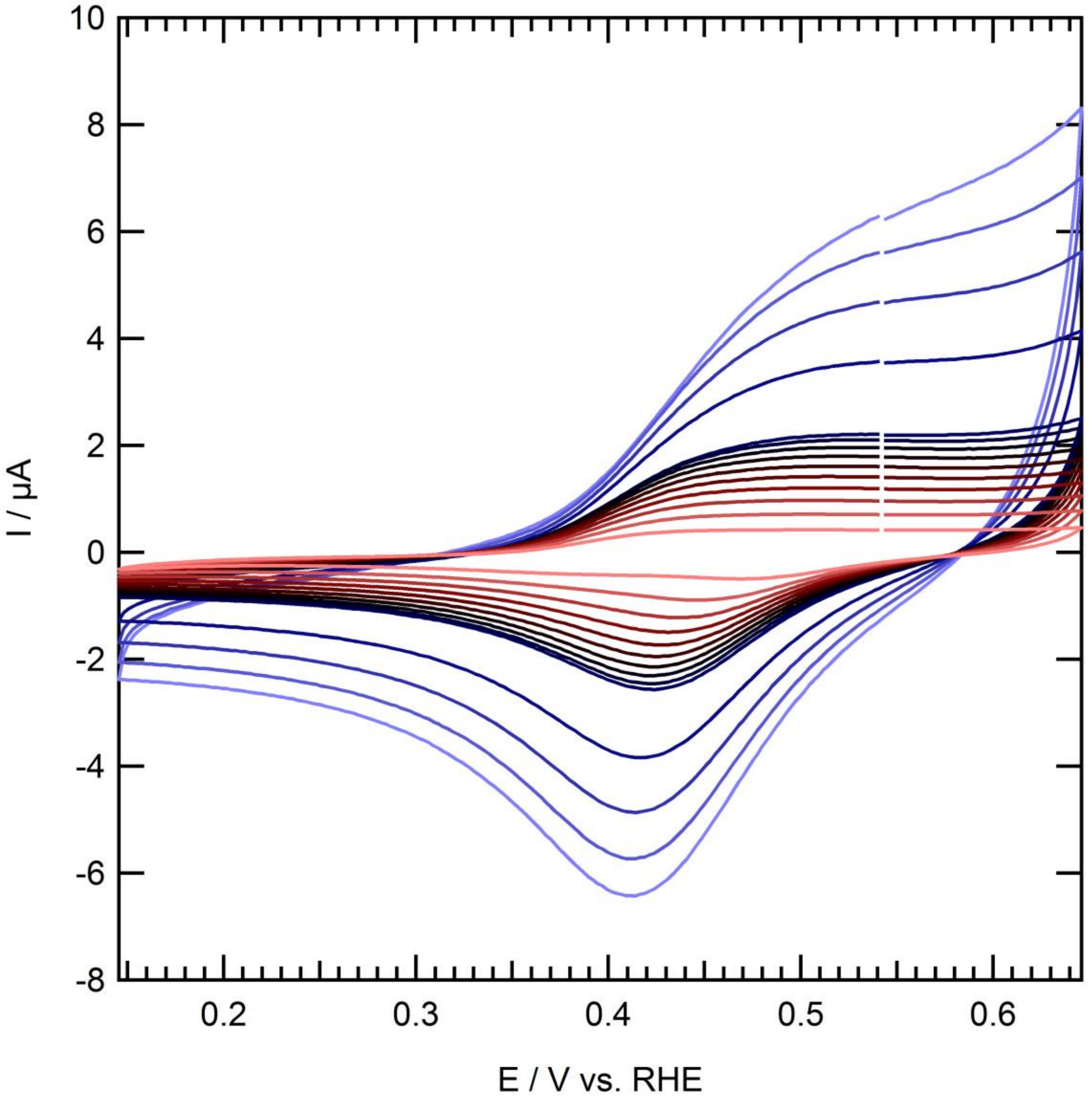

Figure S5: Cyclic voltammograms of a gold electrode under argon in a 1 mM solution of Cu^{II} and 2 mM 1,10-phenanthroline in a 0.1 M phosphate buffer with 0.05 M NaCl acidified to pH 4 at 500 (top blue line) to 10 mV s^{-1} (lowest orange line).

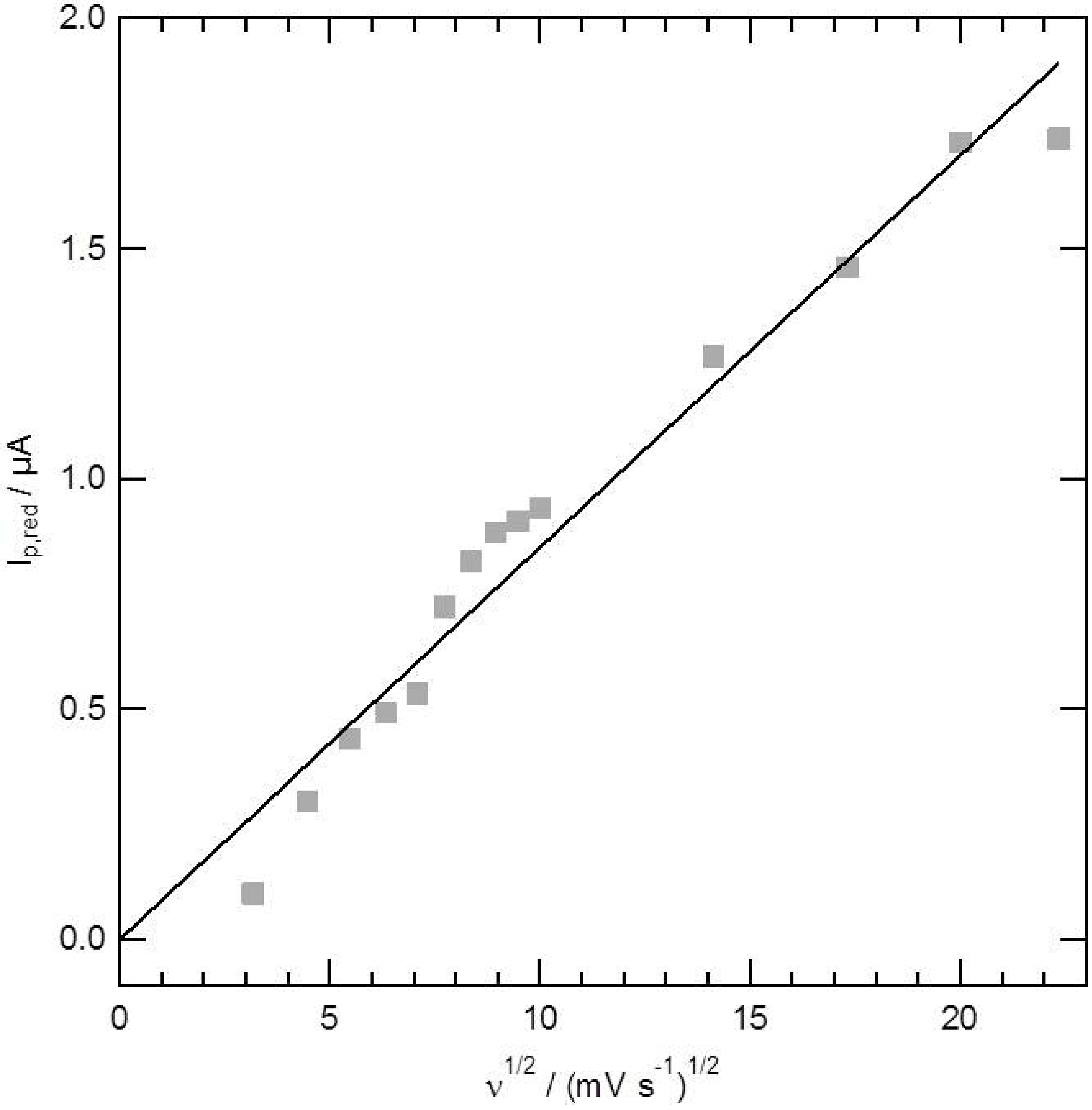

Figure S6: Peak current of the reduction peak *versus* the square root of the scan rate for the investigation of the homogeneity of the *in situ* generated copper-phenanthroline complex.

In the cyclic voltammogram of *in situ* generated copper-DMP complexes from 1 mM DMP and 0.1 mM Cu^{II} in 0.1 M phosphate buffer with 0.05 M NaCl acidified to pH 4 using HCl irreversible behavior of the Cu^{I}/Cu^{II} redox couple is observed (Figure S4b). The separation of the peaks ranges between 470 mV at 10 mV s^{-1} to 660 mV at 500 mV s^{-1} and is thus very large. A large peak separation, and therefore irreversible redox behavior, suggests that copper deposits are being formed.

The oxygen reduction reaction was investigated on a bare gold electrode and with *in situ* generated copper phenanthroline complexes (Figure S7). On a bare gold electrode, oxygen reduction starts at 0.36 V versus RHE. A maximum activity of -20 µA at the vertex potential of 0.24 V versus RHE is observed. Oxygen

reduction with an in situ generated complex from 1 mM 1,10-phenanthroline and 0.1 mM Cu^{II}, a reductive current is observed starting around 0.44 V versus RHE. A starting potential of 0.44 V indicates a high overpotential of 800 mV. A shoulder is observed around 0.35 V, the same potential where oxygen reduction started on a bare gold electrode. The maximum activity of 11 µA is observed at the vertex potential of 0.24 V, which is lower than the activity on the bare gold electrode at the same potential. With oxygen reduction by in situ generated copper complex with 3 mM 1,10-phenanthroline and 1 mM Cu^{II}, an onset potential of 0.41 V versus RHE is observed, similar to the onset potential of the bare gold electrode.

The maximum activity is 13 µA at the vertex potential of 0.24 V, which is slightly higher than the 10:1 complex, but still lower than the bare gold electrode. When the complexes from 1 mM Cu^{II} and 2 mM 1,10-phenanthroline are generated in situ, the onset potential is 0.44 V ver*sus* RHE, which is the same as the 0.1 mM Cu^{II} and 1 mM 1,10-phenanthroline situation. From 0.3 V, the current increases in line with the profile of the bare gold electrode, albeit the current is slightly lower than that of the bare gold. The maximum current is observed at the vertex potential and is 18 µA. The observed current for 1 mM 1,10-phenanthroline with 1 mM Cu^{II} in solution differs greatly from the other measurements. The current starts increasing at 0.45 V and keeps increasing until it reaches a plateau at 0.38 V *versus* RHE. The maximum current observed is 23 µA. The current observed is thus higher than the bare gold electrode and has a higher start potential than the gold electrode. The reductive current indicated the *in situ* generated complex is an active oxygen reduction catalyst.

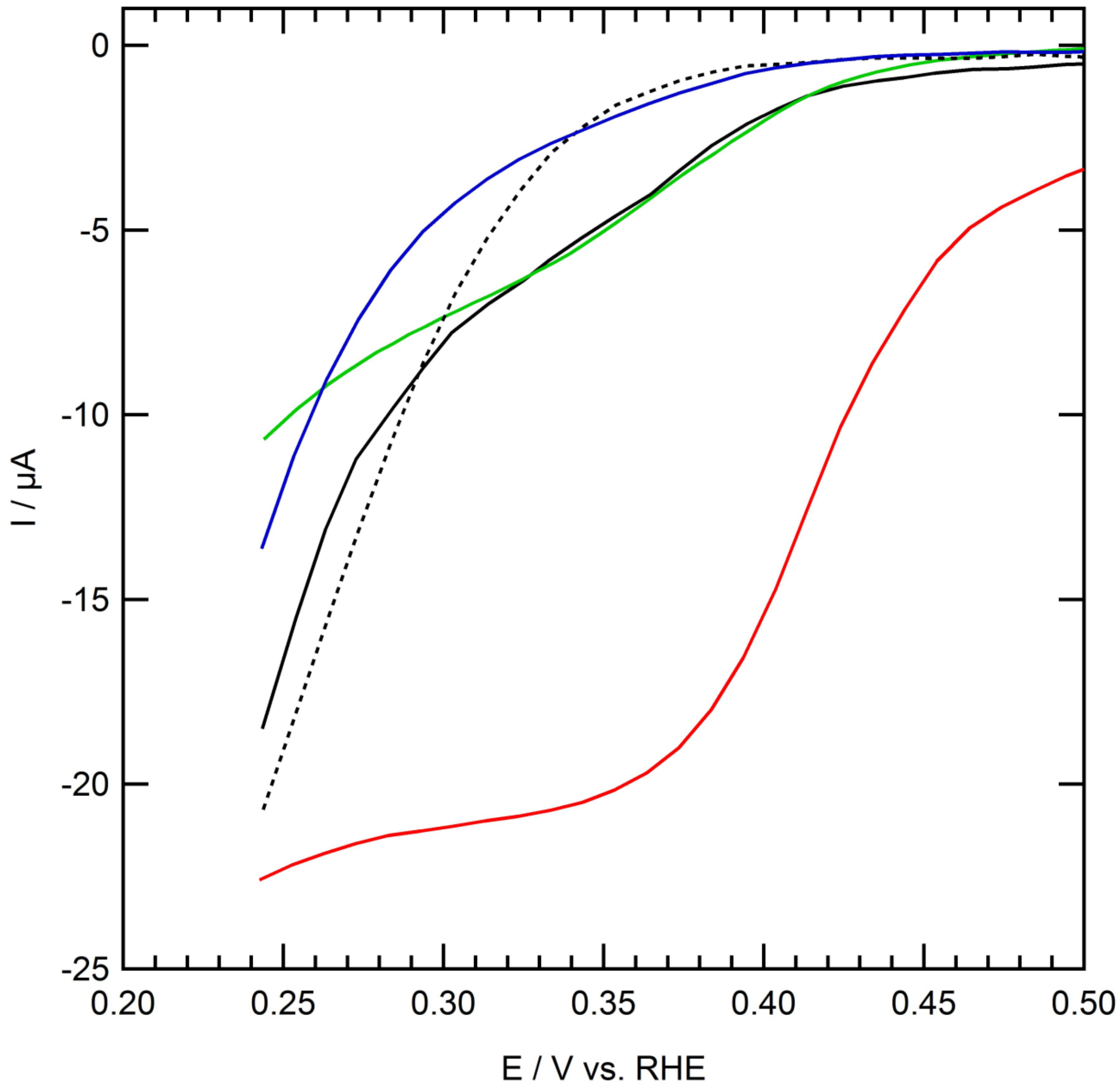

Figure S7: Oxygen reduction at a gold electrode with *in situ* generated copper-phenanthroline complexes with 0.1 mM Cu^{II} and 1 mM 1,10-phenanthroline (green line), 1 mM Cu^{II} and 1 mM 1,10-phenanthroline (red line), 1 mM Cu^{II} and 2 mM 1,10-phenanthroline (black solid line), 1 mM Cu^{II} and 3 mM 1,10-phenanthroline (blue line) and without any catalyst in solution (black dotted line) in a 0.1 M phosphate buffer with 0.05MNaCl acidified to pH 4 using HCl.

EPR SPECTROSCOPY DATA

Since Cu^{II} has 9 d-electrons, one electron will always be unpaired, which makes EPR spectroscopy an excellent tool to determine their structure. Cu^{II} complexes generally adopt two types of geometries: the trigonal bipyramidal and elongated octahedron geometry. Depending on the geometry of the complex, the singly occupied molecular orbital (SOMO) of the complex is either the d_{z^2} or the $d_{x^2-y^2}$ orbital. For the trigonal bipyramidal geometry the SOMO is the d_{z^2} orbital, whereas a Cu^{II} complex in the elongated octahedron geometry has the $d_{x^2-y^2}$ as SOMO. The value of the empirical parameter g, comparable to the chemical shift in NMR spectroscopy, is determined in the x-, y- and z-direction.[1-2] The relative positions of the g-values can be determined by Equation S2, where *i* = *x*,*y* or *z*, *n* is the degree of orbital mixing, λ is the spin-orbit coupling constant, E_0 and E_n are the energies of the ground and the exited states.

$$g_i = g_e \pm \frac{n\lambda}{E_0 - E_n} \quad (S2)$$

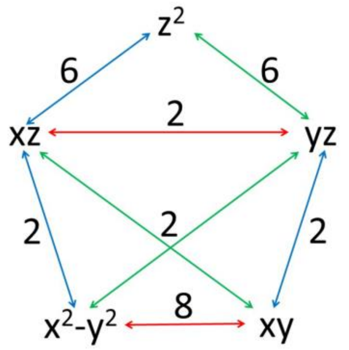

Figure S8: The magic pentagon for the determination of shift of g-values in EPR spectroscopy. The colors represent the direction of the overlap: green is in the x-direction, blue is in the y-direction and red is in the z-direction.

The direction of the shift, represented by ± depends whether the orbital that mix with the SOMO are filled (+) or empty (-). For Cu^{II} complexes, all orbitals except the SOMO are filled and the g-values increase as a result of orbital mixing. The shift of the g-values is thus determined by the mixing factor n, which can be obtained from the so-called "magic pentagon", displayed in Figure S8. The numbers in Figure S8 show the magnitude in which g_i shifts due to orbital mixing along axis i. The colors represent the direction of the overlap: green is in the x-direction, blue is in the y-direction and red is in the z-direction. In a molecule with a mirror plane in x-y and the SOMO is the d_{z^2} orbital, there is no orbital mixing possible in the z-direction, thus $g_z = g_e$. The g_z value in the case of the complex described in this chapter is also called $g_\perp$. Mixing in the x and y-direction is equal resulting in only one g-value, which is called $g_\parallel$. In case the SOMO is the $d_{x^2-y^2}$, orbital mixing occurs in both x, y and z direction. The orbital mixing in x- and y-direction has the same magnitude, resulting in one $g_\parallel$ value. The orbital mixing in z-direction is now larger than the mixing in the xy-plane. This results in a larger $g_\perp$ than $g_\parallel$ (Figure S8). The values for $g_\perp$ and $g_\parallel$ were obtained by fitting the EPR spectra using Win95EPR. The geometry assignment was made on basis of the positions of $g_\perp$ and $g_\parallel$. The structure of the *in situ* generated copper phenanthroline complexes do not differ greatly

if a 1,10-phenanthroline to copper ratio of 2:1 or higher is used (Figure S9 and Table S1). The $g_{\parallel}$ value is either 2.17 or 2.18 and the $g_{\perp}$ values range from 2.01 to 2.04. These values are slightly higher than the values found for copper-phenanthroline complexes in a previous study.[1] The values of 2.17 and ≈2.0 indicate a trigonal bipyramidal structure, with the SOMO being the d_{z^2} orbital. With a one-to-one ratio of copper to phenanthroline, the $g_{\parallel}$ value is 2.05, while the $g_{\perp}$ value is higher and found at 2.25. This is indicative of a complex with an elongated octahedron geometry with the SOMO being the $d_{x^2-y^2}$, orbital. The structure of two similar *in situ* generated 10 to 1 complexes with 2-2'-bipyridine (bipy) and 4,7-dimethyl-1,10-phenanthroline (DMP) ligands were recorded by EPR as well. The g values for 10 to 1 ligand to Cu^{II} complexes with both the DMP and bipy ligands are very similar to the 1,10-phenanthroline complex, indicating the copper complexes are also in the trigonal bipyramidal geometry (table S2).

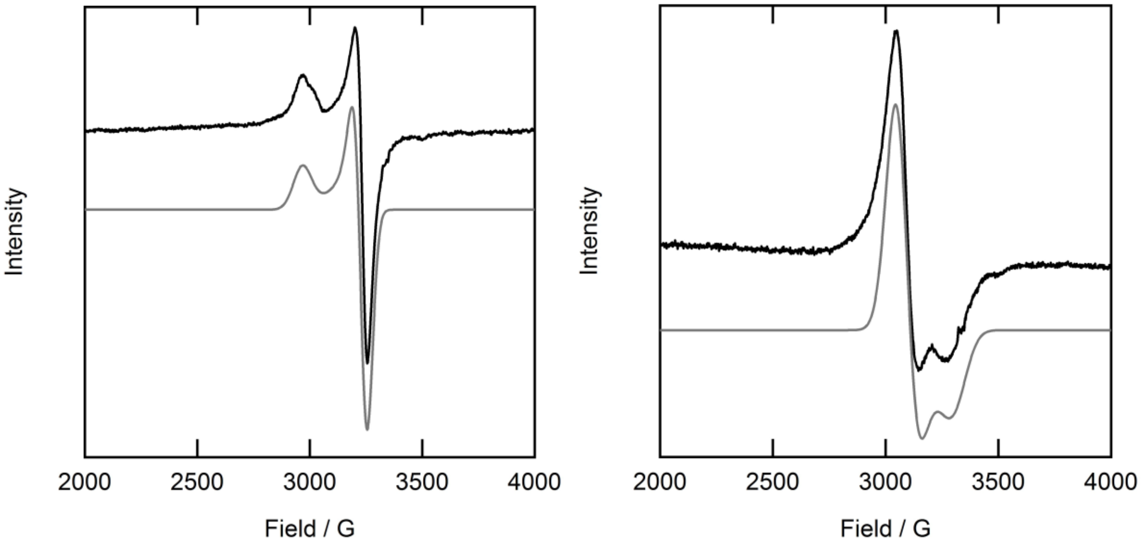

Figure S9: X-band EPR spectra (black lines) and simulations (grey lines) of solutions of 0.1 mM Cu^{II} with a) 0.1 mM and b) 0.2 mM phen in 0.1 M phosphate buffer and 0.05 M NaCl acidified to pH 4 using HCl at 78 K.

Table S1: *EPR parameters of in situ generated Cu^{II} complexes with 1,10-phenanthroline ligands from 0.1 mM Cu^{II} and varying 1,10-phenanthroline concentrations in 0.1 M phosphate buffer, 0.05 M NaCl, acidified to pH 4 using HCl.*

[phen] (mM)	$g_\perp$	$g_\parallel$	SOMO	Geometry
0.1	2.05	2.25	$d_{x^2-y^2}$	Elongated Octahedron
0.2	2.17	2.03	d_{z^2}	Trigonal Bipyramid
0.3	2.17	2.03	d_{z^2}	Trigonal Bipyramid
0.4	2.17	2.02	d_{z^2}	Trigonal Bipyramid
0.5	2.18	2.01	d_{z^2}	Trigonal Bipyramid
0.6	2.18	2.01	d_{z^2}	Trigonal Bipyramid
0.7	2.17	2.04	d_{z^2}	Trigonal Bipyramid
0.8	2.17	2.01	d_{z^2}	Trigonal Bipyramid
0.9	2.18	2.02	d_{z^2}	Trigonal Bipyramid
1.0	2.17	2.03	d_{z^2}	Trigonal Bipyramid

Table S2: *EPR parameters of in situ generated Cu^{II} complexes with different ligands from 0.1 mM Cu^{II} and 1 mM ligand in 0.1 M phosphate buffer, 0.05 M NaCl, acidified to pH 4 using HCl.*

Ligand	$g_\perp$	$g_\parallel$	SOMO	Geometry
Phen	2.17	2.03	d_{z^2}	Trigonal Bipyramid
Bipy	2.17	2.01	d_{z^2}	Trigonal Bipyramid
DMP	2.17	2.01	d_{z^2}	Trigonal Bipyramid

FARADAIC EFFICIENCY DETERMINATION

The Faradaic Efficiency for the diazocoupling reaction is determined as follows:

$$\boldsymbol{Faradaic\ Efficiency} = \frac{\boldsymbol{Charge\ Leading\ to\ Product}}{\boldsymbol{Total\ Charge}} \times \mathbf{100\%}$$

Assumed is that two electrons are necessary. One to reduce $R\text{-}N_2^+$ to $R^\bullet$, and one for the formation of Au-R.

A total amount of 0.9 nmol cm^{-2} and a total charge of 371 µC cm^{-2} were found by EQCM measurements.

This leads to an estimate of 48% for the Faradaic Efficiency determination.

XPS DATA

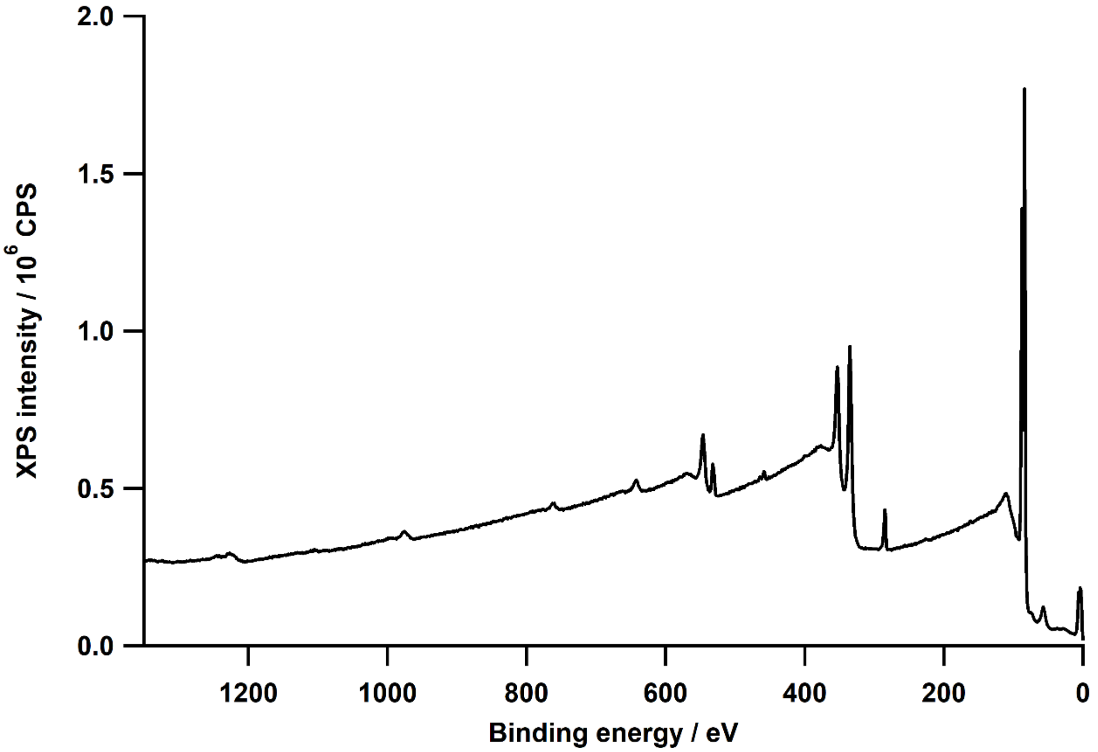

Figure S10. XPS survey bare Au electrode

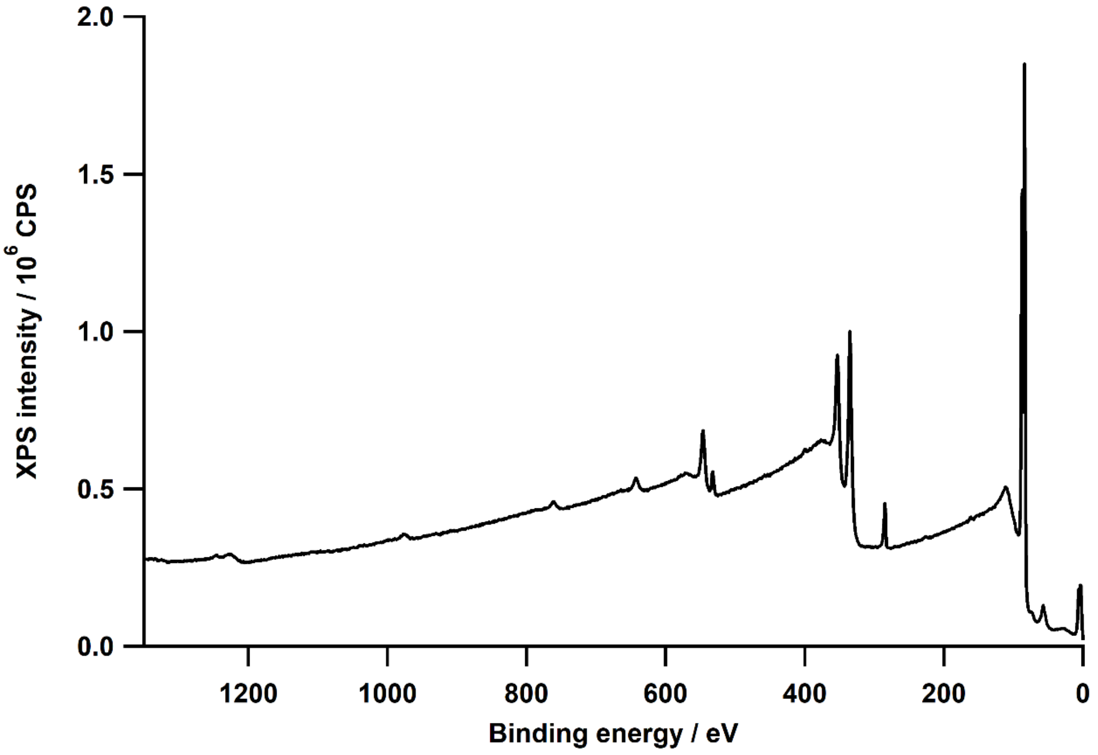

Figure S11. XPS survey spectra of Au|**1**|Cu

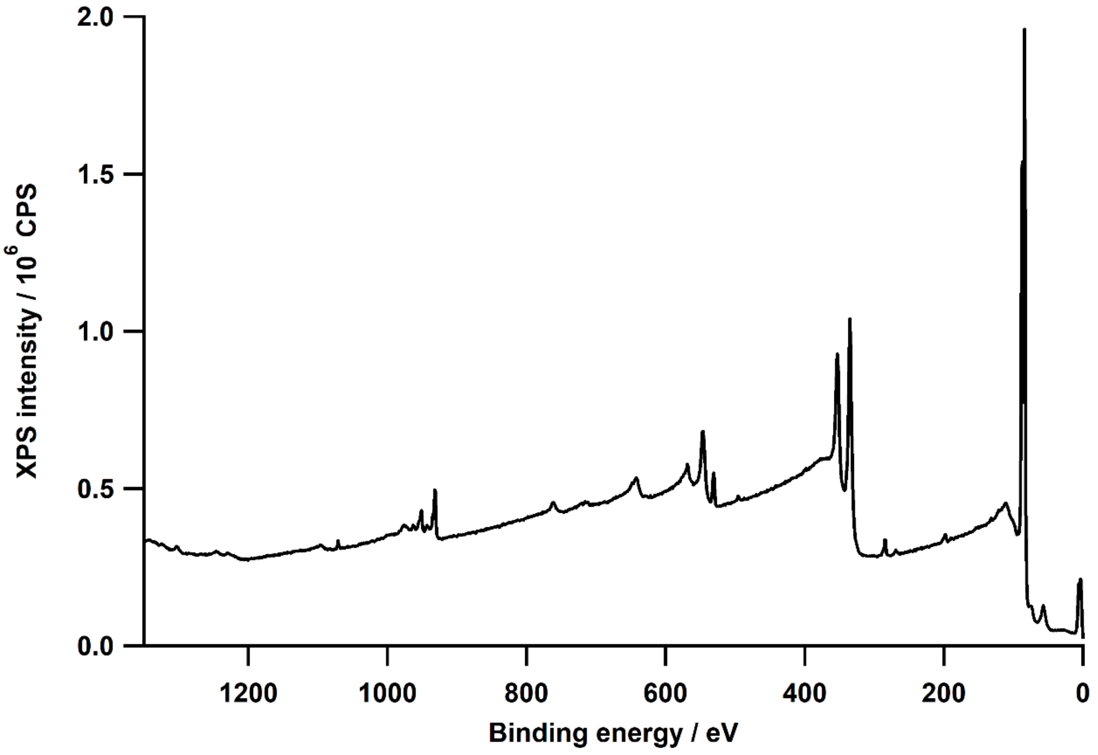

Figure S12. XPS survey spectra of Au|**1**|Cu after 10 cycles ORR

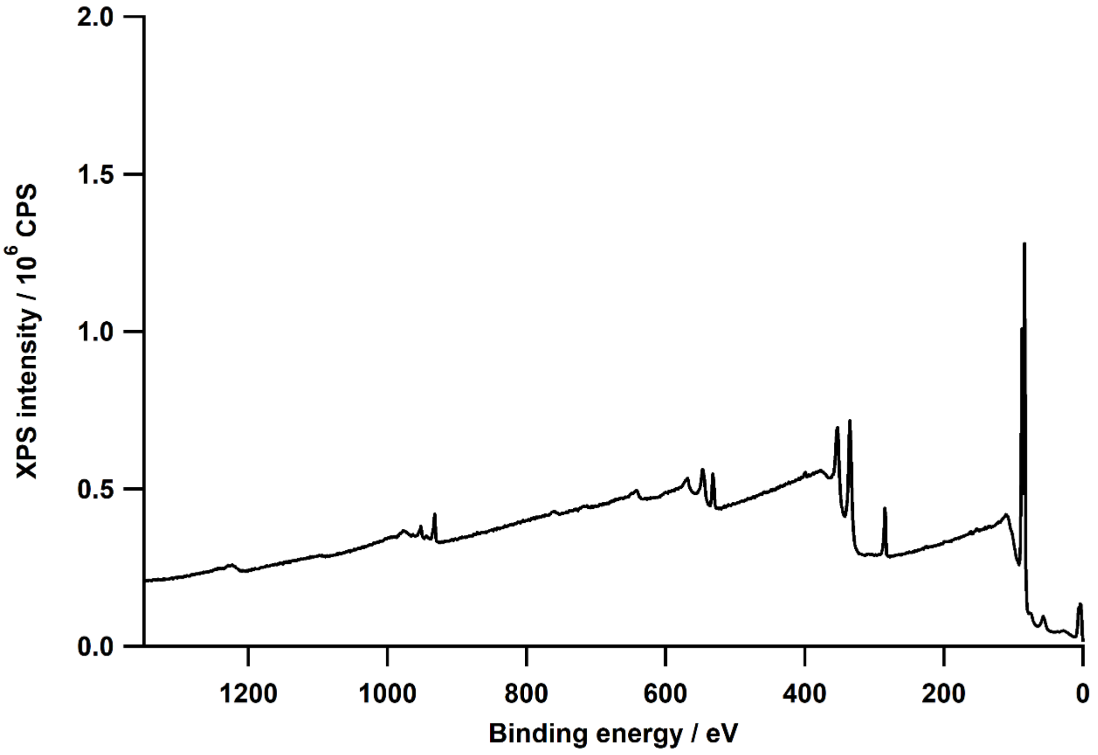

Figure S13. XPS survey spectra of Au|**1**|Cu after 25 cycles ORR

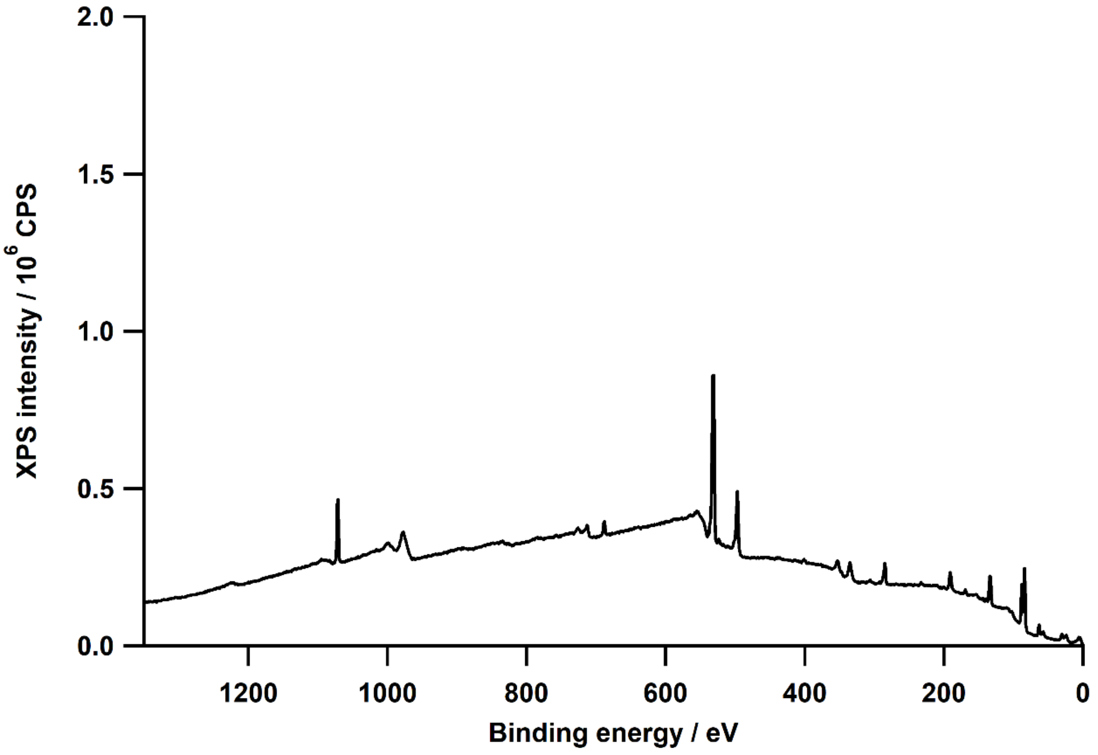

Figure S14. XPS survey spectra of Au|**1**|Cu after 200 cycles ORR

References

1. Garribba, E.; Micera, G., The determination of the geometry of Cu(II) complexes - An EPR spectroscopy experiment. *J Chem Educ* **2006,** *83* (8), 1229-1232.

2. De Bruin, B.; Hetterscheid, D. G. H.; Koekkoek, A. J. J.; Grutzmacher, H., The Organometallic Chemistry of Rh-, Ir-, Pd-, and Pt-Based Radicals: Higher Valent Species. *Prog Inorg Chem* **2007,** *55*, 247-354.
